# Comparative effects of progestin-based combination therapy for endometrial cancer or atypical endometrial hyperplasia: a systematic review and network meta-analysis

**DOI:** 10.3389/fonc.2024.1391546

**Published:** 2024-05-03

**Authors:** Jie Cui, Yue-Chen Zhao, Li-Zhen She, Tie-Jun Wang

**Affiliations:** Department of Radiation Oncology, The Second Hospital of Jilin University, Changchun, Jilin, China

**Keywords:** endometrial cancer, progestin, atypical endometrial hyperplasia, combination therapy, systematic review, meta-analysis

## Abstract

**Objectives:**

The objective of this network meta-analysis is to systematically compare the efficacy of diverse progestin-based combination regimens in treating patients diagnosed with endometrial cancer or atypical endometrial hyperplasia. The primary goal is to discern the optimal combination treatment regimen through a comprehensive examination of their respective effectiveness.

**Methods:**

We systematically searched four prominent databases: PubMed, Web of Science, Embase, and Cochrane Central Register of Controlled Trials, for randomized controlled trials addressing the efficacy of progestins or progestin combinations in the treatment of patients with endometrial cancer or atypical endometrial hyperplasia. The search spanned from the inception of these databases to December 2023. Key outcome indicators encompassed survival indices, criteria for assessing efficacy, as well as pregnancy and relapse rate. This study was registered in PROSPERO (CRD42024496311).

**Results:**

From the 1,558 articles initially retrieved, we included 27 studies involving a total of 5,323 subjects in our analysis. The results of the network meta-analysis revealed that the mTOR inhibitor+megestrol acetate (MA)+tamoxifen regimen secured the top rank in maintaining stable disease (SD) (SUCRA=73.4%) and extending progression-free survival (PFS) (SUCRA=72.4%). Additionally, the progestin combined with tamoxifen regimen claimed the leading position in enhancing the partial response (PR) (SUCRA=75.2%) and prolonging overall survival (OS) (SUCRA=80%). The LNG-IUS-based dual progestin regimen emerged as the frontrunner in improving the complete response (CR) (SUCRA=98.7%), objective response rate (ORR) (SUCRA=99.1%), pregnancy rate (SUCRA=83.7%), and mitigating progression (SUCRA=8.0%) and relapse rate (SUCRA=47.4%). In terms of safety, The LNG-IUS-based dual progestin regimen had the lowest likelihood of adverse events (SUCRA=4.2%), while the mTOR inhibitor regimen (SUCRA=89.2%) and mTOR inbitor+MA+tamoxifen regimen (SUCRA=88.4%) had the highest likelihood of adverse events.

**Conclusions:**

Patients diagnosed with endometrial cancer or atypical endometrial hyperplasia exhibited the most favorable prognosis when undergoing progestin combination therapy that included tamoxifen, mTOR inhibitor, or LNG-IUS. Notably, among these options, the LNG-IUS-based dual progestin regimen emerged as particularly promising for potential application.

**Systematic review registration:**

https://www.crd.york.ac.uk/PROSPERO, identifier CRD42024496311.

## 1 Introduction

Endometrial cancer (EC), arising from the epithelial lining of the uterus, stands as the most prevalent malignant tumor in the female reproductive tract and ranks as the sixth most common cancer in women globally. The disease exhibits an escalating morbidity and mortality rate worldwide. In 2020 alone, there were 417,000 new cases of endometrial cancer, resulting in 97,000 related deaths. The cumulative risk of developing this cancer by the age of 75 is 1%, with a corresponding risk of death at 0.2%. Despite its widespread occurrence, endometrial cancer is generally associated with a favorable prognosis, primarily because, in the majority of cases, the disease is confined to the uterus. The overall 5-year survival rates for endometrial cancer differ by stage: stage I disease boasts an 80% survival rate, stage II at 60%, stage III at 30%, and stage IV at 5%. The incidence of endometrial cancer varies significantly across regions, with a tenfold difference observed globally. The highest incidence rates are documented in Northern America, Europe, Micronesia/Polynesia, and Australia/New Zealand, while the lowest rates are found in most African regions and South Central Asia. In terms of mortality rates, regional disparities are relatively modest. Eastern Europe, Micronesia/Polynesia, the Caribbean, and Northern America report the highest mortality rates (1–4), highlighting the need for increased awareness and targeted interventions in these regions.

Endometrioid neoplastic lesions of the endometrium follow a histological continuum, spanning from endometrial hyperplasia without atypia (EH) to endometrial hyperplasia with atypia (AEH) and well-differentiated endometrial cancer (EC) (5). Broadly categorized into type I and type II, endometrial cancer exhibits distinct characteristics. Type I cancers are primarily associated with obesity-related sequelae, marked by excessive proliferation of endometrial cells. Patients with type I cancer often suffer from hyperestrogenism, hyperlipidemia, diabetes, and anovulatory uterine bleeding-all linked to metabolic syndrome. These type I cancers, mainly highly to moderately differentiated endometrioid tumors, typically originate from atypical endometrial hyperplasia, with at least 90% expressing medium to high levels of estrogen receptors. Conversely, type II cancers are unrelated to metabolic or endocrine disorders and do not stem from endometrial hyperplasia, commonly appearing in non-obese women. This category includes more aggressive histological variants such as clear cell carcinoma, serous carcinoma, or uterine carcinosarcoma (6, 7). Several risk factors contribute to the likelihood of endometrial cancer, with age, race, metabolic syndrome, unopposed estrogen exposure, and genetic predispositions being significant. Unopposed estrogen replacement notably increases the risk of EC by up to 20-fold, with a higher risk associated with prolonged exposure. However, the combined use of progestin significantly reduces this risk (8). Given the dependence of endometrial proliferation on estrogen, well-differentiated tumors are inclined to retain their estrogen and progestin receptors. Additionally, progestin assumes an anti-estrogen role in the endometrium (9).

The standard treatment for endometrial cancer involves surgery, typically with total abdominal hysterectomy and bilateral salpingectomy. In cases with high-risk factors, lymph node dissection followed by individualized adjuvant therapy becomes necessary. Adjuvant treatment strategies for endometrial cancer encompass external-beam pelvic radiotherapy, vaginal brachytherapy, chemotherapy, and the combination of chemotherapy and radiotherapy. Approximately 20% of endometrial cancers occur in premenopausal women, posing a significant concern for maintaining fertility. Surgical treatment, while standard, carries substantial risks for certain patients, particularly those who are morbidly obese or experiencing serious complications (9, 10). Progestin therapy stands as the primary fertility-preserving treatment for young women with atypical endometrial hyperplasia (AEH) and well-differentiated endometrial cancer (EC) (11). Enhancing the efficacy of progestin therapy has become a focal point in addressing the needs of this patient population. For women with AEH who do not intend to bear children, hysterectomy with adnexal removal is strongly recommended, given the approximately 30% risk of developing invasive carcinoma. Conversely, conservative treatment is an option for women who wish to preserve fertility or are at higher surgical risk. These individuals should receive relatively high-dosed progestin therapy, and the use of a progestin-containing intrauterine device is also a viable approach (12). As progestin therapy becomes more prevalent in clinical applications, adverse reactions, including progesterone resistance, thrombosis, and weight gain, are frequently observed, impacting therapeutic efficacy and patient compliance (13). Additionally, a potential consequence of progestin therapy is the reduction in progestin receptor concentration, leading to a shorter duration of response (14). Clinical trials have indicated that progestin combination therapy can mitigate the side effects of long-term progestin use while maintaining its therapeutic benefits. However, there is no consensus on the outcome of progestin combination therapy. For instance, the levonorgestrel-releasing intrauterine system (LNG-IUS) is a device placed in the uterus that increases the concentration of progestin in localized tissues. Fang et al. demonstrated that LNG-IUS combined with oral progestin therapy was more effective in improving menstrual status and endometrial thickness compared to the control group, reducing adverse effects (15). In a 3-year randomized controlled trial, Xu et al. found no difference in treatment efficacy between megestrol acetate (MA) alone, LNG-IUS alone, and MA combined with LNG-IUS (16). Despite these studies, there is still a lack of evidence-based consensus on the efficacy of progestin combination therapy, necessitating further exploration.

Network meta-analyses serve as a valuable tool by amalgamating evidence from both direct and indirect comparisons of different trials, constructing a comprehensive network of treatments. This approach enables researchers to assess the impact of multiple interventions simultaneously for the same condition. Commonly reported outcomes in network meta-analyses include mean ranks or the surface under the cumulative ranking curve (SUCRA), which provides a numerical summary of the distribution to establish hierarchies of treatment comparisons (17, 18). In this study, we employed a network meta-analysis to compare the effectiveness of various progestin combination regimens for patients with endometrial cancer (EC) or atypical endometrial hyperplasia (AEH). The aim is to identify the optimal combination treatment regimen, offering a theoretical reference for the clinical management of AEH or EC patients.

## 2 Materials and methods

### 2.1 Search strategy

The investigators systematically searched PubMed, Web of Science, Embase, and Cochrane Central Register of Controlled Trials for studies published from inception to December 2023. The search strategy was meticulously crafted in accordance with the PICOS tool: (P) Population: patients with endometrial cancer (EC) or atypical endometrial hyperplasia (AEH), (I) Intervention: progestin or progestin combined therapy, (C) Comparator: other conventional treatments (including surgical or irradiation therapy) or progestin, (O) Outcomes: survival index, evaluation criteria for efficacy, pregnancy rate, relapse rate and adverse events, (S) Study type: Randomized Controlled Trials (RCTs). A comprehensive list of the search terms is provided in **Table 1** (using PubMed as an example). Concurrently, Chinese databases such as CNKI were searched, but no literature meeting the inclusion criteria was identified.

**Table 1:** Search strategy on PubMed.

#1	“Progestins”[Mesh]
#2	(((((((((((((((((((((((((((((((((((((Progestagenic Agent) OR (Agent, Progestagenic)) OR (Progestagen)) OR (Progestational Agent)) OR (Agent, Progestational)) OR (Progestational Compound)) OR (Compound, Progestational)) OR (Progestogen)) OR (Progestational Hormone)) OR (Hormone, Progestational)) OR (Gestagens)) OR (Progestagens)) OR (Progestational Hormones)) OR (Progestogens)) OR (Progestational Agents)) OR (Progestational Compounds)) OR (Progestagenic Agents)) OR (Gestagenic Agents)) OR (Progestin)) OR (Gestagen)) OR (Gestagenic Agent)) OR (Agent, Gestagenic)) OR (Progestin Effect)) OR (Effect, Progestin)) OR (Progestogen Effect)) OR (Effect, Progestogen)) OR (Gestagenic Effects)) OR (Effects, Gestagenic)) OR (Gestagen Effect)) OR (Effect, Gestagen)) OR (Gestagen Effects)) OR (Effects, Gestagen)) OR (Gestagenic Effect)) OR (Effect, Gestagenic)) OR (Progestin Effects)) OR (Effects, Progestin)) OR (Progestogen Effects)) OR (Effects, Progestogen)
#3	#1 OR #2
#4	“Endometrial Neoplasms”[Mesh]
#5	((((((((((((((((((Endometrial Neoplasm) OR (Endometrial Neoplasm)) OR (Neoplasms, Endometrial)) OR (Endometrial Carcinoma)) OR (Carcinoma, Endometrial)) OR (Carcinomas, Endometrial)) OR (Endometrial Carcinomas)) OR (Endometrial Cancer)) OR (Cancer, Endometrial)) OR (Cancers, Endometrial)) OR (Endometrial Cancers)) OR (Endometrium Cancer)) OR (Cancer, Endometrium)) OR (Cancers, Endometrium)) OR (Cancer of the Endometrium)) OR (Endometrium Carcinoma)) OR (Endometrium Carcinomas)) OR (Cancer of Endometrium)) OR (Endometrium Cancers)
#6	#4 OR #5
#7	“Endometrial Hyperplasia”[Mesh]
#8	((((((((((((((((((((Endometrial Hyperplasias) OR (Hyperplasia, Endometrial)) OR (Hyperplasias, Endometrial)) OR (Atypical Endometrial Hyperplasia)) OR (Atypical Endometrial Hyperplasias)) OR (Endometrial Hyperplasia, Atypical)) OR (Endometrial Hyperplasias, Atypical)) OR (Hyperplasia, Atypical Endometrial)) OR (Hyperplasias, Atypical Endometrial)) OR (Complex Endometrial Hyperplasia)) OR (Complex Endometrial Hyperplasias)) OR (Endometrial Hyperplasia, Complex)) OR (Endometrial Hyperplasias, Complex)) OR (Hyperplasia, Complex Endometrial)) OR (Hyperplasias, Complex Endometrial)) OR (Simple Endometrial Hyperplasia)) OR (Endometrial Hyperplasia, Simple)) OR (Endometrial Hyperplasias, Simple)) OR (Hyperplasia, Simple Endometrial)) OR (Hyperplasias, Simple Endometrial)) OR (Simple Endometrial Hyperplasias)
#9	#7 OR #8
#10	“Randomized Controlled Trial” [Publication Type]
#11	#3 AND (#6 OR #9) AND #10

### 2.2 Inclusion criteria

The study inclusion criteria encompassed the following key aspects (1): The experimental group involved diverse progestin or progestin combined therapies as interventions for patients (2). The control group received routine treatment or progestin (3). Patients diagnosed with primary endometrial cancer (EC) or atypical endometrial hyperplasia (AEH) were clearly defined and confirmed (4). The study included analyzable data and relevant outcome indices (5). The study design adhered to the Randomized Controlled Trial (RCT) framework.

### 2.3 Exclusion criteria

Studies were excluded based on the following criteria (1): Incomplete or non-extractable data (2). Inconsistencies in diagnostic criteria (3). Studies derived from animal or cell experiments, case reports, scientific experiment plans, protocols, conference abstracts, or correspondence.

### 2.4 Study selection

The literature management process involved importing articles into EndNote 20, followed by a thorough screening and exclusion procedure conducted by two investigators. Initially, the titles of the literature were scrutinized for duplication and the exclusion of non-randomized controlled trial studies, animal or cell experiments, case reports, scientific experiment plans, protocols, conference abstracts, or correspondence. Subsequently, the abstracts of the literature were reviewed by the same investigators to further narrow down the selection. The remaining literature was then meticulously examined in full by the two investigators, who independently identified articles for inclusion, recording the reasons for exclusion. Throughout this elimination process, the two researchers worked independently, and any disparities were resolved through discussion and comparison. If an agreement was reached between the two investigators regarding the inclusion of a particular piece of literature, it was ultimately included. In cases of disagreement, a third investigator made the final decision. This rigorous approach aimed to ensure the meticulous selection of literature for the study.

### 2.5 Data extraction

Data extraction was meticulously conducted using Excel 2010 software. The extracted information encompassed critical details, including author, year of publication, type of disease, sample size, experimental group intervention, control group intervention, mean age, median follow-up, and outcome indicators of the literature. To ensure a standardized and systematic approach, a nine-item, pre-selected data extraction form was employed for recording data, contributing to the comprehensive inclusion of relevant information from the selected studies. This methodical process aimed to uphold consistency and accuracy in the extraction of pertinent data for subsequent analysis. The data reported in the studies we included were obtained based on diagnostic follow-up (hysteroscopic evaluation and curettage biopsy) of the results explored. The outcome indicators were defined as follows (1):. OS was defined as the time from the start of randomization group to death, regardless of cause (2). PFS was defined as the time from the start of randomization group to tumor progression or death from any cause (3). CR was defined as no evidence of hyperplasia or cancerous lesion (4). PR was defined as the improvement in pathological findings (5). ORR was defined as the sum of CR and PR (6). SD was defined as the persistence of disease at initial diagnosis (7). PD was defined as the presence of grade II-III EC, myometrial infiltration or extrauterine lesions in patients with well-differentiated EC and any endometrial malignancy in patients with AEH (8). We included pregnant patients who were in complete response after progestin treatment, with or without assisted reproductive technology (ART) (9). Relapse was defined as the presence of EC or AEH after complete response on progestin therapy (10). Adverse events were defined as any recorded adverse events (AEs) and serious adverse events (SAEs).

### 2.6 Risk of bias of individual studies

Two investigators independently assessed the risk of bias (ROB) using the Cochrane Handbook version 5.1.0 tool. The assessment tool comprises seven domains: (a) randomized sequence generation, (b) concealment of allocation, (c) blinding of participants and personnel, (d) blinding of outcome assessment, (e) incomplete outcome data addressed, (f) freedom from selective reporting bias, and (g) other sources of bias. The risk of bias in each domain is categorized into three levels based on the number of components for which high ROB might exist: high risk (five or more), some concerns (three or four), and low risk (two or less). In cases of disagreement, resolution was achieved through the involvement of a third investigator. Ultimately, a risk of bias graph was generated to visually represent the assessment outcomes. This systematic approach aimed to ensure a rigorous evaluation of potential biases in the included studies.

### 2.7 Statistical analysis

In the intervention studies, all variables were treated as dichotomous and expressed as odds ratios (OR), accompanied by the calculation of the corresponding 95% confidence intervals (CI). Acknowledging the heterogeneity among the studies, this meta-analysis opted for a random-effects model over a fixed-effects model. The analysis was performed using State software (version 15.1) (19). For the Network Meta-Analysis (NMA), Markov chain Monte Carlo simulation chains were employed within a Bayesian-based framework. The nodal method was utilized to quantify consistency between direct and indirect comparisons. The verification of consistency relied on assessing whether the calculated p-value exceeded 0.05 (20).

Network diagrams generated by State software serve to illustrate and elucidate various treatment interventions. Within these diagrams, each node symbolizes a distinct intervention or control condition, while the connecting lines depict direct comparisons between interventions. The size of each node and the thickness of the lines are proportional to the number of patients and relevant studies, respectively (21).

The Surface Under the Cumulative Ranking Curve (SUCRA) metric was employed in this meta-analysis to assess and rank the effectiveness of each treatment. Additionally, it facilitated the identification of the most effective interventions. More precisely, the SUCRA metric comprehensively considers all possible rankings and uncertainties (22). SUCRA values, ranging from 0 to 1, indicate the likelihood of a specific intervention achieving favorable outcomes. A higher SUCRA value suggests a greater probability of the intervention being optimal for the given outcome. A SUCRA value of 1 implies certainty that an intervention is the best, while a value of 0 denotes certainty that an intervention is the least effective. However, differences in SUCRA values do not inherently imply variations in intervention efficacy. A funnel plot was utilized for visual assessment of publication bias, aiding in the identification of potential biases arising from smaller studies (23, 24).

## 3 Results

### 3.1 Study and identification and selection

**Figure 1:**
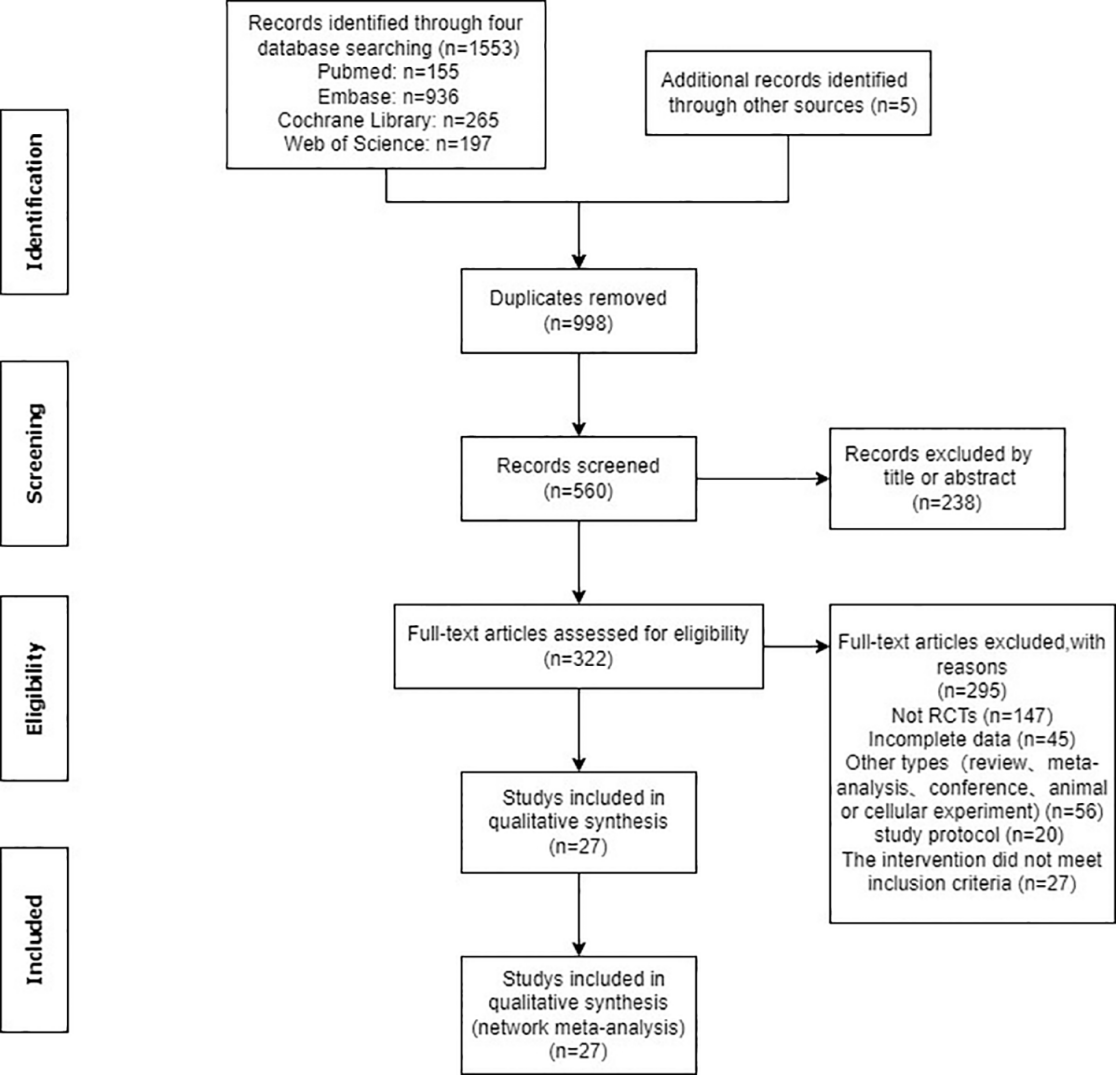
Flow diagram of selection process.

Utilizing specified search strings, a total of 1553 documents were identified in the database. Additionally, five documents were manually retrieved. Following the removal of 998 duplicates, the remaining 560 documents underwent screening based on titles and abstracts, resulting in the exclusion of 238 documents. The remaining 322 papers were thoroughly read and assessed for eligibility, leading to the exclusion of 295 papers due to various reasons, including non-randomized controlled trials (n=147), incomplete data (n=45), other literature types such as conference proceedings (n=56), study protocol (n=20) and interventions not meeting inclusion criteria (n=27). Ultimately, the study included 27 documents (**Figure 1**).

### 3.2 Characteristics of the included studies

A total of 27 randomized controlled trials, encompassing 5,323 patients diagnosed with endometrial cancer or atypical endometrial hyperplasia, were included in this study. These trials spanned the period from 1978 to 2023, with sample sizes ranging from 6 to 1084. The participants’ average age varied between 28.3 and 68.1 years. Among the selected trials, nine were conducted in China (11, 13, 15, 16, 25–29), four in the United States (30–33), three in the United Kingdom (34–36), two in Norway (37, 38), two in Italy (39, 40), and one each in Poland (41), Australia (42), Russia (43), Germany (44), Belgium (45), Iran (46), and the former Soviet Union (47). Of these, 11 trials investigated the effects of progestin-based combination therapy versus progestin alone or other treatments (11, 13, 15, 16, 25, 27, 28, 31, 32, 42, 43), another 11 trials explored the efficacy of progestin alone versus non-progestin treatments (26, 30, 33–35, 37, 40, 41, 44, 45, 47), and 5 trials compared the efficacy of different progestin treatments used alone (29, 36, 38, 39, 46). Most studies incorporated two or more outcome indicators, while a few focused solely on a single survival metric, Overall Survival (OS) (33, 34, 37, 41, 43, 47). **Table 2** provides a summary of the basic characteristics of the included studies.

**Table 2:** Basic characteristics of the included studies.

Author	Year	Disease	Sample size (Total/Int/Ctrl)	Intervention	Control	Mean age ± SD (or range) in years	Median follow-up in months (range)	Outcome
**Bokhman**	1981	EC	276/112/164	HC	general treatment	NR	60 (60)	OS
**Urbanski**	1993	EC	205/100/105	HC	general treatment	56(41-80)	60(60)	OS
**Oza**	2015	EC	95/47/48	MPA	mTOR inhibitor	Int: 66.0(37-80) Ctrl: 65.5(42-81)	3.6(2.7-7.3)	PFS、OS、CR、PR、ORR、SD、PD、Adverse events
**Xu (1)**	2023	AEH	146/48/52,46	MA+LNG-IUS	LNG-IUS MA	Int:33(20-44) Ctrl 1:32(19-44) Ctrl 2:32(23-43)	30.7(9.9–47.5)	CR、ORR、Pregnancy rate、 Relapse rate、Adverse events
**Janda**	2021	EC、AEH	108/33,42/33	LNG-IUS+weight loss LNG-IUS+metformin	LNG-IUS	53(13.9)	6(6)	CR、ORR、Adverse events
**Shan**	2014	AEH	16/8/8	MA+metformin	MA	35(26-43)	3(3)	CR、PR、ORR、SD、PD、 Adverse events
**Kuang**	2021	EC	130/65/65	MA	general treatment	Int:31.13(7.47) Ctrl:29.72(6.61)	45(8-60)	CR、PR、ORR、SD、PD、 Pregnancy rate
**Vishnevsky**	1993	EC	93/37/56	HC+tamoxifen	HC	NR	60(60)	OS
**Vergote**	1989	EC	1084/553/531	HC	general treatment	NR	72(42-132)	OS
**Ørbo**	2016	AEH	153/52,48/53	MPA cycle MPA continue	LNG-IUS	(43-52)	24(24)	CR、ORR、Relapse rate
**Haylock**	1993	EC	218/123/95	MPA	general treatment	Int:65.4 Ctrl:63.6	64(60-120)	OS
**Fleming**	2014	EC	71/21/50	mTOR inhibitor+MA+tamoxifen	mTOR inhibitor	(40-80)	10(6-30)	OS、PFS、CR、PR、ORR、SD、PD、Adverse events
**Pandya**	2001	EC	62/42/20	MA+tamoxifen	MA	Int:65(52-82) Ctrl:68(56-81)	8.6(1.6-46.6)	OS、CR、PR、ORR、SD、PD、 Adverse events
**Fang**	2021	EC、AEH	103/58/45	LNG-IUS+MPA	LNG-IUS	30.12(6.76)	6(6)	CR、PR、ORR、SD、PD、 Pregnancy rate、 Adverse events
**Yuan**	2022	EC	120/60/60	MPA+metformin	MPA	Int:33.73(7.47) Ctrl:35.12(8.41)	NR	CR、PR、ORR、SD、PD、 Pregnancy rate、Adverse events
**Falcone**	2017	EC	28/6/22	MA	LNG-IUS	37(25-40)	92(6-172)	CR 、PR、ORR、SD、PD、 Pregnancy rate、Relapse rate
**Xu (2)**	2023	EC	54/26/28	MA+LNG-IUS	MA	Int:30(25-42) Ctrl:30(21-43)	31.6(3.1-94)	CR、ORR、Pregnancy rate、 Relapse rate、Adverse events
**Rendina**	1984	EC	93/48/45	MPA	tamoxifen	Int:60.4 Ctrl:60.6	28.2(18-40)	CR、PR、ORR、SD、PD、 Relapse rate、Adverse events
**Minckwitz**	2002	EC	388/133/121,134	MPA	tamoxifen general treatment	63(34-75)	56(3-119)	OS、Relapse rate、Adverse events
**COSA-NZ-UK**	1998	EC	1012/505/507	MPA	general treatment	Int:64.4 Ctrl:65.5	64.8(36-120)	OS、PFS、Relapse rate
**Gallos**	2013	AEH	344/25/94	LNG-IUS	MPA	Int:52.7(10.6) Ctrl:48.5(11.6)	87.2(13.2-162)	CR、ORR
**Yang**	2020	EC、AEH	136/70/66	MA+metformin	MA	(18-45)	33.4(26-44)	CR、ORR、Pregnancy rate、 Relapse rate、Adverse events
**Pautier**	2017	EC	73/36/37	MA	STS inhibitor	Int:67.4(8.6) Ctrl:68.1(11.4)	54.1(40.8-66.8)	OS、PFS、CR、PR、ORR、SD、PD、Adverse events
**Malkasian**	1978	EC	35/18/17	MPA	general treatment	Int:63.4(42-75) Ctrl:59.4(43-81)	60(60)	OS
**Kong**	2022	AEH	219/81/138	MPA+metformin	MPA	Int:32(4.58) Ctrl:33.05(5.14)	NR	CR、PR、ORR、SD、PD、 Pregnancy rate
**Mao**	2010	EC	6/4/2	MPA	MA	28.3(26-31)	7.5(3-9)	CR 、PR、ORR、SD、PD、 Pregnancy rate、Relapse rate、Adverse events
**Behnamfar**	2014	AEH	55/27/28	MPA	LNG-IUS	Int:38.6(4.6) Ctrl:38.3(5.1)	3(3)	CR 、PR、ORR、SD、PD、 Adverse events

The terminology used in this study includes control group (Ctrl), intervention group (Int), endometrial cancer (EC), atypical endometrial hyperplasia (AEH), hydroxyprogesterone caproate (HC), megestrol acetate (MA), medroxyprogesterone acetate (MPA), levonorgestrel-releasing intrauterine system (LNG-IUS), general treatment (Surgery, radiotherapy, chemotherapy, and other conventional non-progestogen treatments), mammalian target of rapamycin inhibitor (mTOR inhibitor), steroid sulphatase inhibitor (STS inhibitor), 10 mg of oral MPA administered for 10 days per cycle for 6 months (MPA cycle), and 10 mg of oral MPA administered daily for 6 months (MPA continue). NR denotes data not reported. The study also utilizes abbreviations for outcome measures including overall survival (OS), progression-free survival (PFS), complete response (CR), partial response (PR), objective response rate (ORR), stable disease (SD), progressive disease (PD). Additionally, COSA-NZ-UK refers to the COSA-NZ-UK Endometrial Cancer Study Groups.

### 3.3 Quality assessment of the included studies

Among the randomized controlled trials included in the analysis, 10 studies exhibited a low overall risk of bias, while 15 studies raised some concerns, and only 2 studies presented a high overall risk of bias. The majority of studies (25 out of 27, or 92.3%) seemed to carry a low to moderate risk of bias. 6 studies did not specify how random sequences were generated, and 15 did not provide details about allocation concealment. Given the objective nature of the outcome indicators, blinding of outcome assessment was deemed low risk in all studies. Additionally, 26 studies elucidated the treatment of incomplete data, and 14 studies demonstrated selective reporting. Notably, only eight studies achieved blinding of both participants and assessors to treatment allocation. This limitation arose from the diverse interventions in these studies, involving progestin as well as surgical procedures and radiotherapy, making simultaneous blinding challenging. The included studies did not exhibit evidence of other types of bias (**Supplementary Figure 1**).

### 3.4 Results of network meta-analysis

#### 3.4.1 Survival index

We assessed p-values for consistency and inconsistency in all direct and indirect comparisons related to survival indicators. The findings revealed that all p-values exceeded 0.05, signifying acceptable consistency among the studies (**Supplementary Tables 1**, **2**). The network maps depicting the included interventions are presented in **Supplementary Figures 2A, B**.

##### 3.4.1.1 Overall survival

**Figure 2:**
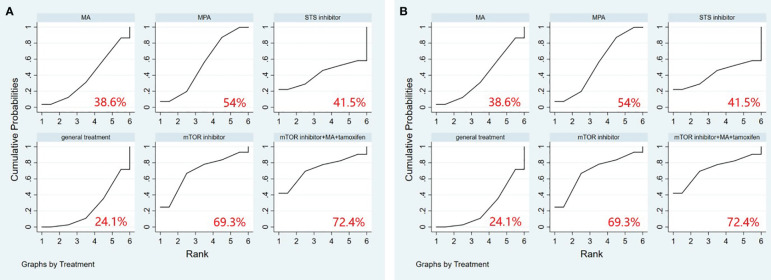
**(A)** SUCRA plot for overall survival (OS), **(B)** SUCRA plot for progression-free survival (PFS). HC, hydroxyprogesterone caproate, MA, megestrol acetate, MPA, medroxyprogesterone acetate, STS, steroid sulphatase, mTOR, mammalian target of rapamycin.

A comprehensive analysis of 10 randomized controlled trials encompassing 13 interventions was conducted to evaluate their impact on Overall Survival (OS). The outcomes from the network meta-analysis, focusing on OS, revealed that none of the interventions demonstrated a clear superiority in terms of OS (**Table 3**). Hydroxyprogesterone caproate (HC) emerged as the most effective intervention for enhancing overall survival, boasting a SUCRA score of 81.4%. Additionally, among all combined treatment regimens, the Hydroxyprogesterone caproate+tamoxifen combination secured the top rank in the probability of improving overall survival, with a SUCRA score of 80% (**Figure 2A**).

**Table 3:** League table on overall survival (OS).

B	C	J	D	G	F	E	A	H	I
**B**	1.35 (0.15,12.39)	0.52 (0.10,2.70)	0.48 (0.07,3.19)	0.43 (0.17,1.12)	0.40 (0.11,1.42)	0.26 (0.02,4.03)	0.16 (0.00,8.63)	0.08 (0.00,2.88)	0.05 (0.00,2.92)
**0.74 (0.08,6.84)**	**C**	0.39 (0.02,6.13)	0.36 (0.02,6.59)	0.32 (0.03,3.59)	0.30 (0.02,3.84)	0.19 (0.01,6.56)	0.12 (0.00,11.40)	0.06 (0.00,4.02)	0.04 (0.00,3.86)
**1.92 (0.37,9.94)**	2.58 (0.16,40.92)	**J**	0.92 (0.11,7.59)	0.83 (0.22,3.14)	0.77 (0.20,2.92)	0.50 (0.03,9.01)	0.31 (0.01,18.43)	0.15 (0.00,5.66)	0.10 (0.00,5.74)
**2.08 (0.31,13.80)**	2.80 (0.15,51.77)	1.08 (0.13,8.94)	**D**	0.90 (0.18,4.62)	0.84 (0.14,5.15)	0.54 (0.07,3.93)	0.33 (0.01,11.14)	0.17 (0.00,7.55)	0.11 (0.00,7.49)
**2.31 (0.89,5.99)**	3.11 (0.28,34.85)	1.21 (0.32,4.56)	1.11 (0.22,5.71)	**G**	0.93 (0.42,2.05)	0.60 (0.05,7.86)	0.37 (0.01,17.79)	0.19 (0.01,5.80)	0.12 (0.00,5.99)
**2.48 (0.71,8.75)**	3.34 (0.26,42.91)	1.29 (0.34,4.90)	1.19 (0.19,7.33)	1.07 (0.49,2.36)	**F**	0.65 (0.04,9.50)	0.40 (0.01,20.68)	0.20 (0.01,5.68)	0.13 (0.00,5.92)
**3.85 (0.25,59.71)**	5.18 (0.15,176.40)	2.01 (0.11,36.29)	1.85 (0.25,13.45)	1.66 (0.13,21.77)	1.55 (0.11,22.82)	**E**	0.62 (0.01,34.73)	0.31 (0.00,22.70)	0.21 (0.00,21.61)
**6.24 (0.12,336.10)**	8.40 (0.09,805.13)	3.25 (0.05,195.01)	3.00 (0.09,100.19)	2.70 (0.06,129.47)	2.51 (0.05,130.59)	1.62 (0.03,91.26)	**A**	0.50 (0.00,89.09)	0.33 (0.00,80.05)
**12.42 (0.35,443.66)**	16.72 (0.25,1124.67)	6.47 (0.18,237.27)	5.97 (0.13,268.73)	5.37 (0.17,167.13)	5.00 (0.18,142.05)	3.23 (0.04,236.10)	1.99 (0.01,352.66)	**H**	0.66 (0.11,4.00)
**18.70 (0.34,1022.39)**	25.18 (0.26,2444.51)	9.75 (0.17,545.28)	8.98 (0.13,604.97)	8.08 (0.17,391.00)	7.53 (0.17,335.87)	4.86 (0.05,509.77)	3.00 (0.01,718.63)	1.51 (0.25,9.07)	**I**

The data in the cells are the OR (95% CI) of the comparison of efficacy between the corresponding interventions. A: STS inhibitor, B: hydroxyprogesterone caproate, C: hydroxyprogesterone caproate+tamoxifen, D: megestrol acetate, E: megestrol acetate+tamoxifen, F: medroxyprogesterone acetate, G: general treatment, H: mTOR inhibitor, I: mTOR inhibitor+megestrol acetate+tamoxifen, J: tamoxifen.

##### 3.4.1.2 Progression-free survival

A comprehensive analysis was conducted on 5 randomized controlled trials involving 6 interventions to assess their impact on Progression-Free Survival (PFS). Among these interventions, Medroxyprogesterone acetate (MPA) [OR=1.44, 95% CI= (1.05, 1.98)] demonstrated superiority over general treatment in comparison to the control group (**Table 4**). According to the Surface Under the Cumulative Ranking Curve (SUCRA), the combination of mTOR inhibitor, megestrol acetate (MA), and tamoxifen exhibited the most significant influence on PFS, achieving a SUCRA score of 72.4% (**Figure 2B**).

**Table 4:** League table on progression-free survival (PFS).

F	E	B	C	A	D
**F**	0.83 (0.14,4.90)	0.28 (0.01,10.96)	0.22 (0.00,51.92)	0.23 (0.01,9.91)	0.19 (0.00,7.71)
**1.21** **(0.20,7.17)**	**E**	0.33 (0.01,8.39)	0.27 (0.00,46.67)	0.27 (0.01,7.69)	0.23 (0.01,5.91)
**3.63** **(0.09,144.56)**	3.00 (0.12,75.51)	**B**	0.80 (0.01,45.24)	0.82 (0.35,1.93)	0.69 (0.51,0.95)
**4.56** **(0.02,1081.07)**	3.77 (0.02,663.12)	1.26 (0.02,71.42)	**C**	1.03 (0.02,53.33)	0.87 (0.02,48.94)
**4.43** **(0.10,194.87)**	3.66 (0.13,103.19)	1.22 (0.52,2.88)	0.97 (0.02,50.36)	**A**	0.85 (0.38,1.88)
**5.24** **(0.13,211.22)**	4.32 (0.17,110.55)	1.44 (1.05,1.98)	1.15 (0.02,64.42)	1.18 (0.53,2.63)	**D**

Important results will be presented with a yellow background. A: megestrol acetate,B: medroxyprogesterone acetate, C: STS inhibitor, D: general treatment, E: mTOR inhibitor, F: mTOR inhibitor+ megestrol acetate+tamoxifen.

#### 3.4.2 Evaluation criteria for the efficacy

All p-values comparing the involved studies were assessed for both consistency and inconsistency. In the non-consistency test, all p-values exceeded 0.05. Similarly, in the consistency test, the majority of p-values were greater than 0.05. In summary, these results suggest that the consistency across studies was acceptable (**Supplementary Tables 3**–**7**). The network maps illustrating the included interventions are presented in **Supplementary Figures 3A–E**.

##### 3.4.2.1 Complete response

**Figure 3:**
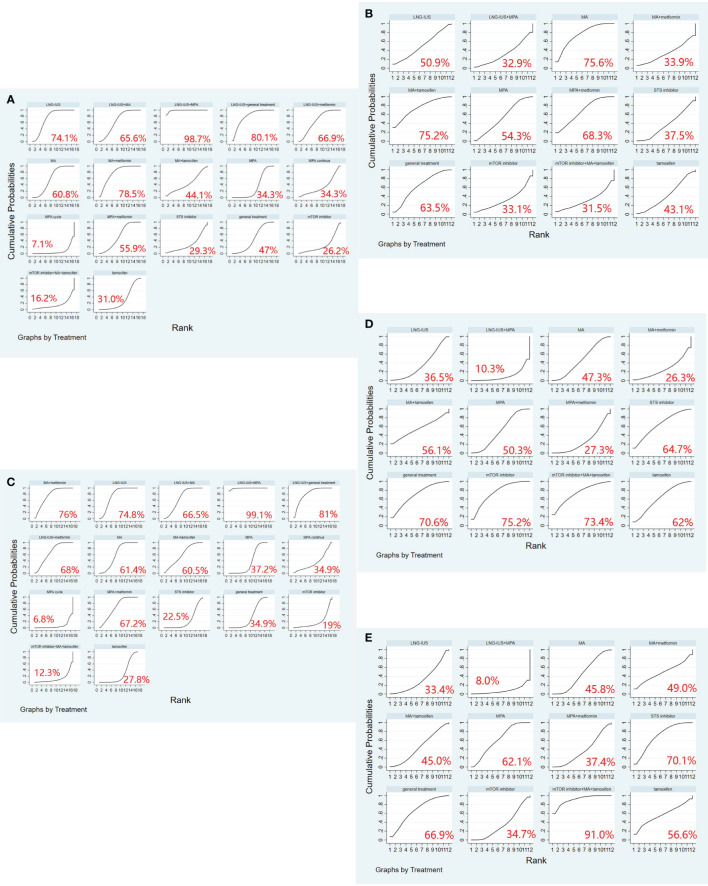
**(A)** SUCRA plot for complete response (CR), **(B)** SUCRA plot for partial response (PR), **(C)** SUCRA plot for objective response rate (ORR), **(D)** SUCRA plot for stable disease (SD), **(E)** SUCRA plot for progressive disease (PD). MA, megestrol acetate, MPA, medroxyprogesterone acetate, LNG-IUS, levonorgestrel-releasing intrauterine system, STS, steroid sulphatase, mTOR, mammalian target of rapamycin.

A total of 19 randomized controlled trials involving 17 interventions were analyzed for their impact on Complete Response (CR). The findings indicated that the LNG-IUS+MPA group [OR=14.75, 95% CI=(4.58, 47.52)], LNG-IUS+general treatment group [OR=4.20, 95% CI=(1.25, 14.13)], MA+metformin group [OR=3.75, 95% CI=(1.03, 13.68)], LNG-IUS group [OR=3.23, 95% CI=(1.64, 6.37)], and MPA+metformin group [OR=1.93, 95% CI=(1.01, 3.71)] were more effective than the MPA group in increasing the number of CR post-treatment. Additionally, when compared with the tamoxifen group, the LNG-IUS+MPA group [OR=19.18, 95% CI=(3.73, 98.69)], LNG-IUS+general treatment group [OR=5.46, 95% CI=(1.03, 28.99)], and LNG-IUS group [OR=4.20, 95% CI=(1.11, 15.92)] outperformed the MPA group. Ultimately, compared to the MPA cycle group, the LNG-IUS+MPA group [OR=220.92, 95% CI=(11.00, 4437.09)], LNG-IUS+general treatment group [OR=62.88, 95% CI=(3.08, 1285.05)], MA+metformin group [OR=56.08, 95% CI=(2.63, 1196.31)], LNG-IUS group [OR=48.37, 95% CI=(2.81, 831.86)], LNG-IUS+metformin group [OR=41.92, 95% CI=(2.10, 835.53)], LNG-IUS+MA group [OR=38.92, 95% CI=(1.96, 771.98)], MA group [OR=34.54, 95% CI=(1.76, 678.22)], MPA+metformin group [OR=28.92, 95% CI=(1.44, 578.99)], general treatment group [OR=23.48, 95% CI=(1.10, 500.67)], and MPA continue group [OR=8.41, 95% CI=(2.08, 34.06)] were all superior to the MPA group (**Table 5**). The LNG-IUS+MPA group ranked first in the SUCRA probability rankings (SUCRA=98.7%) (**Figure 3A**).

**Table 5:** League table on complete response (CR).

C	D	G	A	E	B	F	L	N	H	I	J	Q	M	O	P	K
**C**	0.28 (0.07,1.14)	0.25 (0.06,1.11)	0.22 (0.08,0.57)	0.19 (0.05,0.72)	0.18 (0.05,0.66)	0.16 (0.04,0.57)	0.13 (0.03,0.50)	0.11 (0.02,0.46)	0.07 (0.00,1.62)	0.07 (0.02,0.22)	0.04 (0.00,0.94)	0.05 (0.01,0.27)	0.03 (0.00,0.85)	0.02 (0.00,0.67)	0.01 (0.00,0.65)	0.00 (0.00,0.09)
**3.51 (0.88,14.04)**	**D**	0.89 (0.20,4.04)	0.77 (0.28,2.10)	0.67 (0.26,1.72)	0.62 (0.16,2.41)	0.55 (0.14,2.09)	0.46 (0.12,1.82)	0.37 (0.08,1.69)	0.25 (0.01,5.79)	0.24 (0.07,0.80)	0.13 (0.01,3.36)	0.18 (0.03,0.97)	0.11 (0.00,3.04)	0.08 (0.00,2.39)	0.02 (0.00,2.33)	0.02 (0.00,0.33)
**3.94 (0.90,17.24)**	1.12 (0.25,5.08)	**G**	0.86 (0.28,2.67)	0.75 (0.17,3.22)	0.69 (0.25,1.91)	0.62 (0.30,1.25)	0.52 (0.12,2.20)	0.42 (0.15,1.14)	0.29 (0.02,5.25)	0.27 (0.07,0.98)	0.15 (0.01,3.92)	0.21 (0.04,1.16)	0.12 (0.01,2.80)	0.09 (0.00,2.76)	0.03 (0.00,2.67)	0.02 (0.00,0.38)
**4.57 (1.76,11.84)**	1.30 (0.48,3.55)	1.16 (0.38,3.58)	**A**	0.87 (0.34,2.19)	0.80 (0.32,2.00)	0.71 (0.30,1.72)	0.60 (0.23,1.53)	0.49 (0.16,1.50)	0.33 (0.02,6.37)	0.31 (0.16,0.61)	0.17 (0.01,3.71)	0.24 (0.06,0.90)	0.14 (0.01,3.39)	0.10 (0.00,2.67)	0.03 (0.00,2.71)	0.02 (0.00,0.36)
**5.27 (1.39,19.92)**	1.50 (0.58,3.87)	1.34 (0.31,5.76)	1.15 (0.46,2.92)	**E**	0.93 (0.25,3.41)	0.82 (0.23,2.96)	0.69 (0.18,2.59)	0.56 (0.13,2.41)	0.38 (0.02,8.48)	0.36 (0.11,1.13)	0.20 (0.01,4.92)	0.27 (0.05,1.39)	0.16 (0.01,4.46)	0.11 (0.00,3.51)	0.04 (0.00,3.43)	0.02 (0.00,0.48)
**5.68 (1.52,21.22)**	1.62 (0.42,6.28)	1.44 (0.52,3.97)	1.24 (0.50,3.09)	1.08 (0.29,3.96)	**B**	0.89 (0.43,1.83)	0.74 (0.20,2.72)	0.60 (0.22,1.66)	0.41 (0.02,7.59)	0.38 (0.13,1.18)	0.22 (0.01,5.27)	0.30 (0.06,1.47)	0.17 (0.01,4.05)	0.12 (0.00,3.74)	0.04 (0.00,3.67)	0.03 (0.00,0.51)
**6.40 (1.75,23.36)**	1.82 (0.48,6.92)	1.62 (0.80,3.30)	1.40 (0.58,3.37)	1.21 (0.34,4.35)	1.13 (0.55,2.32)	**F**	0.84 (0.24,2.97)	0.68 (0.34,1.38)	0.46 (0.03,7.81)	0.43 (0.15,1.28)	0.24 (0.01,5.88)	0.33 (0.07,1.62)	0.19 (0.01,4.20)	0.14 (0.00,4.16)	0.04 (0.00,4.10)	0.03 (0.00,0.57)
**7.64 (2.00,29.15)**	2.17 (0.55,8.62)	1.94 (0.45,8.27)	1.67 (0.65,4.29)	1.45 (0.39,5.44)	1.35 (0.37,4.92)	1.19 (0.34,4.24)	**L**	0.81 (0.19,3.46)	0.55 (0.03,12.23)	0.52 (0.27,0.99)	0.29 (0.01,7.16)	0.40 (0.11,1.49)	0.23 (0.01,6.44)	0.17 (0.01,4.45)	0.05 (0.00,4.51)	0.03 (0.00,0.69)
**9.41 (2.15,41.13)**	2.68 (0.59,12.12)	2.39 (0.88,6.49)	2.06 (0.67,6.35)	1.79 (0.41,7.68)	1.66 (0.60,4.55)	1.47 (0.73,2.98)	1.23 (0.29,5.25)	**N**	0.68 (0.04,12.53)	0.64 (0.17,2.33)	0.36 (0.01,9.35)	0.49 (0.09,2.76)	0.29 (0.01,6.69)	0.20 (0.01,6.59)	0.06 (0.00,6.37)	0.04 (0.00,0.91)
**13.80 (0.62,308.68)**	3.93 (0.17,89.34)	3.50 (0.19,64.44)	3.02 (0.16,58.20)	2.62 (0.12,58.14)	2.43 (0.13,44.90)	2.16 (0.13,36.37)	1.81 (0.08,39.92)	1.47 (0.08,26.97)	**H**	0.94 (0.05,19.28)	0.53 (0.01,37.09)	0.72 (0.03,18.30)	0.42 (0.01,27.24)	0.30 (0.00,24.91)	0.09 (0.00,19.83)	0.06 (0.00,3.78)
**14.75 (4.58,47.52)**	4.20 (1.25,14.13)	3.75 (1.03,13.68)	3.23 (1.64,6.37)	2.80 (0.89,8.84)	2.60 (0.85,7.97)	2.31 (0.78,6.83)	1.93 (1.01,3.71)	1.57 (0.43,5.72)	1.07 (0.05,22.04)	**I**	0.56 (0.02,12.92)	0.77 (0.24,2.42)	0.45 (0.02,11.66)	0.32 (0.01,8.05)	0.10 (0.00,8.30)	0.07 (0.00,1.24)
**26.27 (1.06,648.70)**	7.48 (0.30,187.66)	6.67 (0.26,174.23)	5.75 (0.27,122.90)	4.99 (0.20,122.21)	4.63 (0.19,112.95)	4.11 (0.17,99.30)	3.44 (0.14,84.67)	2.79 (0.11,72.92)	1.90 (0.03,134.44)	1.78 (0.08,40.98)	**J**	1.37 (0.05,38.62)	0.80 (0.01,66.72)	0.57 (0.01,51.18)	0.18 (0.00,40.22)	0.12 (0.03,0.48)
**19.18 (3.73,98.69)**	5.46 (1.03,28.99)	4.87 (0.86,27.47)	4.20 (1.11,15.92)	3.64 (0.72,18.46)	3.38 (0.68,16.79)	3.00 (0.62,14.54)	2.51 (0.67,9.39)	2.04 (0.36,11.49)	1.39 (0.05,35.34)	1.30 (0.41,4.09)	0.73 (0.03,20.58)	**Q**	0.58 (0.02,18.44)	0.42 (0.01,12.75)	0.13 (0.00,12.49)	0.09 (0.00,2.01)
**32.88 (1.17,921.64)**	9.36 (0.33,266.46)	8.35 (0.36,195.11)	7.20 (0.30,175.61)	6.24 (0.22,173.68)	5.79 (0.25,135.92)	5.14 (0.24,110.89)	4.30 (0.16,119.30)	3.49 (0.15,81.66)	2.38 (0.04,154.60)	2.23 (0.09,57.89)	1.25 (0.01,104.47)	1.71 (0.05,54.17)	**M**	0.71 (0.01,69.74)	0.22 (0.00,54.04)	0.15 (0.00,10.72)
**46.17 (1.49,1427.22)**	13.14 (0.42,412.45)	11.72 (0.36,378.91)	10.11 (0.37,273.08)	8.76 (0.29,269.01)	8.13 (0.27,247.30)	7.22 (0.24,217.01)	6.04 (0.22,162.42)	4.91 (0.15,158.59)	3.35 (0.04,278.75)	3.13 (0.12,78.76)	1.76 (0.02,158.00)	2.41 (0.08,73.83)	1.40 (0.01,137.51)	**O**	0.32 (0.02,6.38)	0.21 (0.00,16.26)
**146.27 (1.53,14011.69)**	41.63 (0.43,4034.03)	37.13 (0.37,3679.13)	32.03 (0.37,2774.48)	27.76 (0.29,2645.43)	25.77 (0.27,2437.94)	22.87 (0.24,2145.38)	19.15 (0.22,1652.47)	15.55 (0.16,1540.07)	10.60 (0.05,2227.76)	9.91 (0.12,815.33)	5.57 (0.02,1246.40)	7.63 (0.08,726.25)	4.45 (0.02,1069.49)	3.17 (0.16,64.06)	**P**	0.66 (0.00,131.51)
**220.92 (11.00,4437.09)**	62.88 (3.08,1285.05)	56.08 (2.63,1196.31)	48.37 (2.81,831.86)	41.92 (2.10,835.53)	38.92 (1.96,771.98)	34.54 (1.76,678.22)	28.92 (1.44,578.99)	23.48 (1.10,500.67)	16.01 (0.26,969.80)	14.97 (0.80,278.93)	8.41 (2.08,34.06)	11.52 (0.50,266.50)	6.72 (0.09,484.20)	4.79 (0.06,372.34)	1.51 (0.01,300.00)	**K**

Important results will be presented with a yellow background. A: LNG-IUS, B: LNG-IUS+megestrol acetate, C: LNG-IUS+medroxyprogesterone acetate, D: LNG-IUS+general treatment, E: LNG-IUS+metformin, F: megestrol acetate, G: megestrol acetate+metformin, H: megestrol acetate+tamoxifen, I: medroxyprogesterone acetate, J: medroxyprogesterone acetate continue, K: medroxyprogesterone acetate cycle, L: medroxyprogesterone acetate+metformin, M: STS inhibitor, N: general treatment, O: mTOR inhibitor, P: mTOR inhibitor+megestrol acetate+tamoxifen, Q: tamoxifen.

##### 3.4.2.2 Partial response

A total of 13 randomized controlled trials involving 12 interventions were analyzed for their impact on Partial Response (PR). The outcomes of the network meta-analysis, with Partial Response (PR) as the outcome, revealed that the MA group [OR=4.07, 95% CI=(1.02, 16.31)] demonstrated superiority in increasing the number of individuals in PR compared to the STS inhibitor group (**Table 6**). Megestrol acetate (MA) emerged as the most effective intervention to enhance overall survival among all interventions (SUCRA=75.6%). Within combined treatment regimens, the MA+tamoxifen group secured the top position in the probability ranking for improving overall survival (SUCRA=75.2%) (**Figure 3B**).

**Table 6:** League table on partial response (PR).

C	E	G	I	F	A	L	H	D	J	B	K
**C**	1.13 (0.26,4.94)	0.66 (0.02,20.61)	0.71 (0.34,1.47)	0.45 (0.02,13.01)	0.35 (0.01,9.56)	0.31 (0.01,10.26)	0.25 (0.06,0.98)	0.15 (0.01,3.77)	0.14 (0.00,15.16)	0.17 (0.00,5.96)	0.12 (0.00,16.46)
**0.88** **(0.20,3.84)**	**E**	0.58 (0.01,24.58)	0.63 (0.12,3.24)	0.39 (0.01,15.60)	0.31 (0.01,11.54)	0.27 (0.01,12.18)	0.22 (0.03,1.64)	0.13 (0.00,4.58)	0.13 (0.00,16.77)	0.15 (0.00,7.04)	0.11 (0.00,18.04)
**1.51** **(0.05,47.07)**	1.71 (0.04,72.12)	**G**	1.07 (0.03,36.10)	0.67 (0.35,1.30)	0.53 (0.02,15.02)	0.47 (0.15,1.47)	0.37 (0.01,15.12)	0.23 (0.00,25.40)	0.21 (0.01,5.79)	0.26 (0.01,9.34)	0.19 (0.01,6.83)
**1.41** **(0.68,2.91)**	1.59 (0.31,8.22)	0.93 (0.03,31.26)	**I**	0.63 (0.02,19.76)	0.50 (0.02,14.54)	0.43 (0.01,15.54)	0.35 (0.07,1.65)	0.22 (0.01,5.74)	0.20 (0.00,22.54)	0.24 (0.01,9.02)	0.18 (0.00,24.43)
**2.25** **(0.08,65.64)**	2.55 (0.06,101.10)	1.49 (0.77,2.87)	1.60 (0.05,50.42)	**F**	0.79 (0.03,20.91)	0.69 (0.27,1.77)	0.55 (0.01,21.19)	0.34 (0.00,36.05)	0.32 (0.01,8.04)	0.38 (0.01,13.06)	0.28 (0.01,9.56)
**2.83** **(0.10,76.54)**	3.21 (0.09,118.67)	1.87 (0.07,52.67)	2.01 (0.07,58.90)	1.26 (0.05,33.18)	**A**	0.87 (0.03,26.27)	0.69 (0.02,24.85)	0.43 (0.00,42.95)	0.40 (0.00,39.81)	0.48 (0.13,1.82)	0.35 (0.00,43.39)
**3.24 (0.10,107.52)**	3.67 (0.08,163.92)	2.14 (0.68,6.75)	2.30 (0.06,82.37)	1.44 (0.56,3.68)	1.14 (0.04,34.38)	**L**	0.79 (0.02,34.38)	0.50 (0.00,57.05)	0.46 (0.02,13.25)	0.55 (0.01,21.27)	0.40 (0.01,15.57)
**4.07** **(1.02,16.31)**	4.62 (0.61,34.87)	2.70 (0.07,109.89)	2.90 (0.61,13.86)	1.81 (0.05,69.68)	1.44 (0.04,51.51)	1.26 (0.03,54.45)	**H**	0.62 (0.02,20.45)	0.58 (0.00,75.54)	0.69 (0.02,31.51)	0.51 (0.00,81.35)
**6.54 (0.27,160.97)**	7.41 (0.22,251.67)	4.33 (0.04,475.51)	4.65 (0.17,124.15)	2.91 (0.03,305.35)	2.31 (0.02,229.26)	2.02 (0.02,232.70)	1.60 (0.05,52.66)	**D**	0.93 (0.00,267.60)	1.11 (0.01,133.30)	0.81 (0.00,280.21)
**7.03 (0.07,748.81)**	7.97 (0.06,1064.26)	4.65 (0.17,125.16)	5.00 (0.04,563.22)	3.13 (0.12,78.76)	2.48 (0.03,245.60)	2.17 (0.08,62.48)	1.73 (0.01,224.86)	1.08 (0.00,309.27)	**J**	1.20 (0.01,142.82)	0.88 (0.21,3.68)
**5.88 (0.17,205.81)**	6.66 (0.14,312.48)	3.89 (0.11,141.24)	4.18 (0.11,157.50)	2.62 (0.08,89.39)	2.08 (0.55,7.86)	1.82 (0.05,70.12)	1.44 (0.03,65.58)	0.90 (0.01,107.72)	0.84 (0.01,99.86)	**B**	0.73 (0.00,107.93)
**8.03 (0.06,1062.34)**	9.10 (0.06,1495.47)	5.32 (0.15,193.09)	5.71 (0.04,797.09)	3.58 (0.10,122.20)	2.84 (0.02,349.61)	2.48 (0.06,95.86)	1.97 (0.01,316.29)	1.23 (0.00,422.97)	1.14 (0.27,4.81)	1.37 (0.01,201.63)	**K**

Important results will be presented with a yellow background. A: LNG-IUS, B: LNG-IUS+medroxyprogesterone acetate, C: megestrol acetate, D: megestrol acetate+metformin, E: megestrol acetate+tamoxifen, F: medroxyprogesterone acetate, G: medroxyprogesterone acetate+metformin, H: STS inhibitor, I: general treatment, J:mTOR inhibitor, K: mTOR inhibitor+megestrol acetate+tamoxifen, L: tamoxifen.

##### 3.4.2.3 Objective response rate

In total, 19 randomized controlled trials involving 17 interventions were analyzed for their effects on Objective Response Rate (ORR). The results revealed that, in comparison to the tamoxifen group, the LNG-IUS+MPA group [OR=27.17, 95%CI=(5.41, 136.41)], MA+metformin [OR=5.21, 95%CI=(1.12, 24.26)], LNG-IUS+general treatment group [OR=6.44, 95%CI=(1.48, 28.08)], LNG-IUS group [OR=4.95, 95%CI=(1.69, 14.52)], LNG-IUS+metformin group [OR=4.29, 95%CI=(1.04, 17.77)], and MPA+metformin group [OR=4.10, 95%CI=(1.40, 11.97)] demonstrated superior efficacy. Similarly, compared to the Steroid Sulphatase Inhibitor (STS) group, the LNG-IUS+MA group [OR=5.95, 95%CI=(1.27, 27.96)], LNG-IUS+MPA group [OR=40.55, 95%CI=(5.37, 306.32)], MA group [OR=5.28, 95%CI=(1.34, 20.73)], MA+metformin group [OR=7.77, 95%CI=(1.67, 36.19)], LNG-IUS+treatment group [OR=9.61, 95%CI=(1.42, 65.00)], and LNG-IUS group [OR=7.39, 95%CI=(1.46, 37.56)] exhibited superiority. Furthermore, in comparison to the MPA cycle group, the LNG-IUS+MA group [OR=38.92, 95%CI=(1.96, 771.97)], LNG-IUS+MPA group [OR=265.28, 95%CI=(12.09, 5822.25)], MA group [OR=34.54, 95%CI=(1.76, 678.21)], MA+metformin group [OR=50.86, 95%CI=(2.39, 1083.85)], LNG-IUS+general treatment group [OR=62.88, 95%CI=(3.08, 1285.05)], LNG-IUS group [OR=48.37, 95%CI=(2.81, 831.86)], LNG-IUS+metformin group [OR=41.92, 95%CI=(2.10, 835.53)], MPA+metformin group [OR=39.99, 95%CI=(1.99, 804.54)], MA+tamoxifen group [OR=32.51, 95%CI=(1.24, 850.80)], and MPA continue group [OR=8.41, 95%CI=(2.08, 34.06)] surpassed the tamoxifen group (**Table 7**). Lastly, compared to the LNG-IUS+MPA group, the LNG-IUS+MA group [OR=0.15, 95%CI=(0.03,0.66)] did not exhibit a significant advantage in improving ORR. According to the SUCRA, the LNG-IUS+MPA group ranked first (SUCRA=99.1%) in the probability ranking of different interventions for improving ORR (**Figure 3C**).

**Table 7:** League table on objective response rate (ORR).

C	D	G	A	E	B	F	L	N	H	I	J	Q	M	O	P	K
**C**	6.82 (1.51,30.84)	0.89 (0.43,1.83)	1.31 (0.48,3.58)	1.62 (0.42,6.28)	1.24 (0.50,3.09)	1.08 (0.29,3.96)	1.03 (0.28,3.80)	0.35 (0.12,1.06)	0.84 (0.18,3.83)	0.38 (0.13,1.18)	0.22 (0.01,5.27)	0.25 (0.06,1.01)	0.17 (0.04,0.79)	0.07 (0.00,1.88)	0.04 (0.00,1.48)	0.03 (0.00,0.51)
**0.15 (0.03,0.66)**	**D**	0.13 (0.03,0.58)	0.19 (0.04,1.00)	0.24 (0.05,1.14)	0.18 (0.05,0.61)	0.16 (0.03,0.72)	0.15 (0.03,0.70)	0.05 (0.01,0.28)	0.12 (0.02,0.91)	0.06 (0.01,0.22)	0.03 (0.00,0.85)	0.04 (0.01,0.18)	0.02 (0.00,0.19)	0.01 (0.00,0.30)	0.01 (0.00,0.24)	0.00 (0.00,0.08)
**1.13 (0.55,2.32)**	7.68 (1.73,34.06)	**G**	1.47 (0.73,2.97)	1.82 (0.48,6.92)	1.40 (0.58,3.37)	1.21 (0.34,4.35)	1.16 (0.32,4.15)	0.40 (0.17,0.91)	0.94 (0.25,3.59)	0.43 (0.15,1.28)	0.24 (0.01,5.88)	0.28 (0.07,1.11)	0.19 (0.05,0.74)	0.08 (0.00,2.10)	0.05 (0.00,1.65)	0.03 (0.00,0.57)
**0.77 (0.28,2.10)**	5.22 (1.00,27.08)	0.68 (0.34,1.37)	**A**	1.24 (0.27,5.59)	0.95 (0.31,2.93)	0.82 (0.19,3.54)	0.79 (0.18,3.38)	0.27 (0.09,0.80)	0.64 (0.14,2.90)	0.29 (0.08,1.07)	0.17 (0.01,4.31)	0.19 (0.04,0.89)	0.13 (0.03,0.60)	0.06 (0.00,1.54)	0.03 (0.00,1.20)	0.02 (0.00,0.42)
**0.62 (0.16,2.41)**	4.22 (0.88,20.24)	0.55 (0.14,2.09)	0.81 (0.18,3.66)	**E**	0.77 (0.28,2.10)	0.67 (0.26,1.72)	0.64 (0.16,2.55)	0.22 (0.05,1.05)	0.52 (0.08,3.43)	0.24 (0.07,0.80)	0.13 (0.01,3.36)	0.16 (0.04,0.68)	0.10 (0.02,0.70)	0.04 (0.00,1.21)	0.03 (0.00,0.94)	0.02 (0.00,0.33)
**0.80 (0.32,2.00)**	5.48 (1.65,18.26)	0.71 (0.30,1.72)	1.05 (0.34,3.24)	1.30 (0.48,3.55)	**B**	0.87 (0.34,2.19)	0.83 (0.32,2.15)	0.28 (0.09,0.95)	0.67 (0.14,3.33)	0.31 (0.16,0.61)	0.17 (0.01,3.71)	0.20 (0.07,0.59)	0.14 (0.03,0.69)	0.06 (0.00,1.34)	0.03 (0.00,1.06)	0.02 (0.00,0.36)
**0.93 (0.25,3.41)**	6.33 (1.39,28.91)	0.82 (0.23,2.96)	1.21 (0.28,5.22)	1.50 (0.58,3.87)	1.15 (0.46,2.92)	**F**	0.95 (0.25,3.62)	0.33 (0.07,1.50)	0.78 (0.12,4.94)	0.36 (0.11,1.13)	0.20 (0.01,4.92)	0.23 (0.06,0.96)	0.16 (0.02,1.01)	0.07 (0.00,1.77)	0.04 (0.00,1.39)	0.02 (0.00,0.48)
**0.97 (0.26,3.60)**	6.63 (1.43,30.86)	0.86 (0.24,3.10)	1.27 (0.30,5.47)	1.57 (0.39,6.30)	1.21 (0.46,3.15)	1.05 (0.28,3.98)	**L**	0.34 (0.08,1.58)	0.81 (0.13,5.18)	0.37 (0.19,0.74)	0.21 (0.01,5.20)	0.24 (0.08,0.71)	0.16 (0.03,1.06)	0.07 (0.00,1.62)	0.04 (0.00,1.28)	0.03 (0.00,0.50)
**2.83 (0.94,8.50)**	19.29 (3.51,105.98)	2.51 (1.10,5.74)	3.70 (1.25,10.95)	4.57 (0.95,21.99)	3.52 (1.05,11.75)	3.05 (0.67,13.97)	2.91 (0.63,13.32)	**N**	2.36 (0.49,11.41)	1.09 (0.28,4.26)	0.61 (0.02,16.42)	0.71 (0.14,3.51)	0.48 (0.10,2.35)	0.20 (0.01,5.84)	0.12 (0.00,4.56)	0.07 (0.00,1.60)
**1.20 (0.26,5.49)**	8.16 (1.10,60.47)	1.06 (0.28,4.05)	1.56 (0.34,7.10)	1.93 (0.29,12.82)	1.49 (0.30,7.38)	1.29 (0.20,8.21)	1.23 (0.19,7.83)	0.42 (0.09,2.04)	**H**	0.46 (0.08,2.58)	0.26 (0.01,8.19)	0.30 (0.04,2.04)	0.20 (0.03,1.36)	0.09 (0.00,2.90)	0.05 (0.00,2.24)	0.03 (0.00,0.80)
**2.60 (0.85,7.97)**	17.72 (4.45,70.52)	2.31 (0.78,6.83)	3.40 (0.93,12.38)	4.20 (1.25,14.13)	3.23 (1.64,6.37)	2.80 (0.89,8.84)	2.67 (1.36,5.25)	0.92 (0.23,3.59)	2.17 (0.39,12.17)	**I**	0.56 (0.02,12.92)	0.65 (0.28,1.50)	0.44 (0.08,2.50)	0.19 (0.01,4.01)	0.11 (0.00,3.21)	0.07 (0.00,1.24)
**4.63 (0.19,112.95)**	31.55 (1.18,846.49)	4.11 (0.17,99.30)	6.05 (0.23,157.87)	7.48 (0.30,187.66)	5.75 (0.27,122.90)	4.99 (0.20,122.21)	4.76 (0.19,117.61)	1.64 (0.06,43.94)	3.87 (0.12,122.44)	1.78 (0.08,40.98)	**J**	1.16 (0.05,29.80)	0.78 (0.02,24.91)	0.33 (0.00,26.78)	0.20 (0.00,19.64)	0.12 (0.03,0.48)
**3.99 (0.99,16.10)**	27.17 (5.41,136.41)	3.54 (0.90,13.90)	5.21 (1.12,24.26)	6.44 (1.48,28.08)	4.95 (1.69,14.52)	4.29 (1.04,17.77)	4.10 (1.40,11.97)	1.41 (0.28,6.97)	3.33 (0.49,22.59)	1.53 (0.67,3.53)	0.86 (0.03,22.10)	**Q**	0.67 (0.10,4.64)	0.29 (0.01,6.88)	0.17 (0.01,5.45)	0.10 (0.00,2.14)
**5.95 (1.27,27.96)**	40.55 (5.37,306.32)	5.28 (1.34,20.73)	7.77 (1.67,36.19)	9.61 (1.42,65.00)	7.39 (1.46,37.56)	6.41 (0.99,41.64)	6.11 (0.94,39.73)	2.10 (0.43,10.40)	4.97 (0.73,33.70)	2.29 (0.40,13.11)	1.29 (0.04,41.15)	1.49 (0.22,10.33)	**M**	0.43 (0.01,14.59)	0.25 (0.01,11.24)	0.15 (0.01,4.05)
**13.85 (0.53,361.40)**	94.42 (3.28,2718.92)	12.29 (0.48,316.96)	18.10 (0.65,503.15)	22.38 (0.83,603.65)	17.22 (0.75,396.78)	14.92 (0.57,393.32)	14.23 (0.62,327.76)	4.90 (0.17,139.98)	11.57 (0.34,388.89)	5.33 (0.25,114.02)	2.99 (0.04,239.85)	3.47 (0.15,83.11)	2.33 (0.07,79.12)	**O**	0.59 (0.15,2.38)	0.36 (0.01,24.59)
**23.44 (0.68,813.43)**	159.79 (4.20,6072.58)	20.81 (0.61,714.09)	30.64 (0.83,1126.84)	37.88 (1.06,1355.09)	29.14 (0.94,902.33)	25.25 (0.72,884.55)	24.09 (0.78,745.43)	8.28 (0.22,312.80)	19.58 (0.45,858.80)	9.02 (0.31,261.00)	5.06 (0.05,503.83)	5.88 (0.18,188.40)	3.94 (0.09,174.58)	1.69 (0.42,6.82)	**P**	0.60 (0.01,52.02)
**38.92 (1.96,771.97)**	265.28 (12.09,5822.25)	34.54 (1.76,678.21)	50.86 (2.39,1083.85)	62.88 (3.08,1285.05)	48.37 (2.81,831.86)	41.92 (2.10,835.53)	39.99 (1.99,804.54)	13.75 (0.63,302.27)	32.51 (1.24,850.80)	14.97 (0.80,278.93)	8.41 (2.08,34.06)	9.76 (0.47,204.34)	6.54 (0.25,173.24)	2.81 (0.04,194.07)	1.66 (0.02,143.37)	**K**

Important results will be displayed with a yellow or blue background. A: megestrol acetate+metformin, B: LNG-IUS, C: LNG-IUS+megestrol acetate, D: LNG-IUS+medroxyprogesterone acetate, E: LNG-IUS+general treatment, F: LNG-IUS+metformin, G: megestrol acetate, H: megestrol acetate+tamoxifen, I: medroxyprogesterone acetate, J: medroxyprogesterone acetate continue, K: medroxyprogesterone acetate cycle, L: medroxyprogesterone acetate+metformin, M: STS inhibitor, N: general treatment, O: mTOR inhibitor, P: mTOR inhibitor+megestrol acetate+tamoxifen, Q: tamoxifen.

##### 3.4.2.4 Stable disease

In total, 13 randomized controlled trials involving 12 interventions were analyzed for their effects on Stable Disease (SD). The results of the network meta-analysis with SD as the outcome revealed that the mTOR inhibitor group [OR=20.62, 95%CI=(1.15, 369.19)] outperformed the LNG-IUS+MPA group in increasing the number of SD after treatment (**Table 8**). According to the SUCRA, the mTOR inhibitor was identified as the most effective intervention for improving overall survival among all interventions (SUCRA=75.2%). Additionally, among all combined treatment regimens, the mTOR inhibitor+MA+tamoxifen group achieved the top rank in the probability ranking of improving overall survival (SUCRA=73.4%) (**Figure 3D**).

**Table 8:** League table on stable disease (SD).

J	K	I	H	L	E	F	C	A	G	D	B
**J**	1.02 (0.22,4.71)	0.80 (0.03,24.65)	0.64 (0.02,19.85)	0.57 (0.07,4.51)	0.51 (0.00,52.10)	0.37 (0.08,1.67)	0.34 (0.02,7.58)	0.21 (0.02,2.22)	0.16 (0.02,1.09)	0.11 (0.00,5.78)	0.05 (0.00,0.87)
**0.98 (0.21,4.57)**	**K**	0.79 (0.02,33.70)	0.63 (0.01,27.13)	0.56 (0.04,7.38)	0.50 (0.00,65.74)	0.37 (0.04,3.14)	0.34 (0.01,10.70)	0.21 (0.01,3.45)	0.16 (0.01,1.84)	0.11 (0.00,7.60)	0.05 (0.00,1.25)
**1.25 (0.04,38.42)**	1.27 (0.03,54.15)	**I**	0.80 (0.10,6.52)	0.72 (0.02,21.30)	0.64 (0.02,26.92)	0.47 (0.02,10.18)	0.43 (0.10,1.88)	0.26 (0.01,5.39)	0.20 (0.01,5.65)	0.14 (0.01,2.41)	0.06 (0.00,1.91)
**1.56 (0.05,48.20)**	1.58 (0.04,67.90)	1.25 (0.15,10.15)	**H**	0.89 (0.03,26.72)	0.80 (0.02,33.76)	0.58 (0.03,12.79)	0.54 (0.12,2.38)	0.33 (0.02,6.77)	0.25 (0.01,7.09)	0.18 (0.01,3.03)	0.08 (0.00,2.39)
**1.74 (0.22,13.70)**	1.77 (0.14,23.12)	1.40 (0.05,41.52)	1.12 (0.04,33.44)	**L**	0.89 (0.01,88.54)	0.65 (0.16,2.69)	0.60 (0.03,12.73)	0.37 (0.04,3.68)	0.28 (0.04,1.79)	0.20 (0.00,9.77)	0.08 (0.00,1.45)
**1.96 (0.02,200.12)**	1.99 (0.02,260.35)	1.57 (0.04,66.34)	1.26 (0.03,53.41)	1.12 (0.01,111.94)	**E**	0.73 (0.01,58.36)	0.67 (0.02,21.06)	0.42 (0.01,31.52)	0.31 (0.00,30.02)	0.22 (0.00,14.99)	0.10 (0.00,9.87)
**2.67 (0.60,11.95)**	2.71 (0.32,23.16)	2.14 (0.10,46.70)	1.72 (0.08,37.64)	1.53 (0.37,6.33)	1.36 (0.02,108.58)	**F**	0.92 (0.06,13.78)	0.57 (0.09,3.46)	0.42 (0.13,1.44)	0.31 (0.01,11.47)	0.13 (0.01,1.53)
**2.90 (0.13,63.99)**	2.95 (0.09,93.10)	2.33 (0.53,10.18)	1.86 (0.42,8.26)	1.67 (0.08,35.34)	1.48 (0.05,46.24)	1.09 (0.07,16.26)	**C**	0.62 (0.04,8.52)	0.46 (0.02,9.32)	0.33 (0.03,3.70)	0.14 (0.01,3.18)
**4.72 (0.45,49.44)**	4.79 (0.29,79.27)	3.78 (0.19,77.08)	3.03 (0.15,62.12)	2.71 (0.27,26.97)	2.41 (0.03,182.86)	1.77 (0.29,10.79)	1.63 (0.12,22.52)	**A**	0.75 (0.08,7.14)	0.54 (0.02,19.13)	0.23 (0.04,1.22)
**6.30 (0.91,43.43)**	6.40 (0.54,75.33)	5.04 (0.18,143.72)	4.04 (0.14,115.77)	3.61 (0.56,23.43)	3.21 (0.03,309.97)	2.36 (0.70,7.98)	2.17 (0.11,43.84)	1.33 (0.14,12.71)	**G**	0.72 (0.02,34.02)	0.31 (0.02,5.06)
**8.71 (0.17,438.69)**	8.85 (0.13,595.10)	6.98 (0.41,117.54)	5.59 (0.33,94.78)	5.00 (0.10,244.25)	4.45 (0.07,296.18)	3.26 (0.09,121.90)	3.00 (0.27,33.31)	1.85 (0.05,65.18)	1.38 (0.03,65.12)	**D**	0.42 (0.01,21.69)
**20.62 (1.15,369.19)**	20.94 (0.80,549.61)	16.52 (0.52,519.70)	13.23 (0.42,418.56)	11.83 (0.69,203.33)	10.52 (0.10,1092.04)	7.71 (0.65,90.84)	7.10 (0.31,160.29)	4.37 (0.82,23.33)	3.27 (0.20,54.28)	2.37 (0.05,121.48)	**B**

Important results will be presented with a yellow background. A: LNG-IUS, B: LNG-IUS+medroxyprogesterone acetate, C: megestrol acetate, D: megestrol acetate+metformin, E: megestrol acetate+tamoxifen, F: medroxyprogesterone acetate, G: medroxyprogesterone acetate+metformin, H: STS inhibitor, I: general treatment, J: mTOR inhibitor, K: mTOR inhibitor+megestrol acetate+tamoxifen, L: tamoxifen.

##### 3.4.2.5 Progressive disease

In total, 13 randomized controlled trials involving 12 interventions were analyzed for their effects on Progressive Disease (PD). The results of the network meta-analysis indicated that there were more instances of disease progression in the mTOR inhibitor+MA+tamoxifen group [OR=22.37, 95%CI=(1.75, 285.42)] compared to the MPA+metformin group. Moreover, in comparison to the mTOR inhibitor group, both the mTOR inhibitor+MA+tamoxifen group [OR=24.50, 95%CI=(2.78, 216.27)] and the MPA group [OR=2.64, 95%CI=(1.11, 6.29)] showed an increased likelihood of disease progression after treatment. Furthermore, when compared to the LNG-IUS+MPA group, the mTOR inhibitor+MA+tamoxifen group [OR=288.57, 95%CI=(2.77, 30039.79)] exhibited a higher risk of disease progression after treatment (**Table 9**). In the SUCRA analysis, the LNG-IUS+MPA group ranked last (SUCRA=8.0%) in the probability ranking of different interventions leading to disease progression (**Figure 3E**).

**Table 9:** League table on progressive disease (PD).

K	H	I	F	L	D	C	E	G	J	A	B
**K**	0.16 (0.00,8.85)	0.15 (0.00,9.10)	0.11 (0.01,1.12)	0.11 (0.00,11.26)	0.07 (0.00,18.68)	0.07 (0.00,3.35)	0.07 (0.00,3.80)	0.04 (0.00,0.57)	0.04 (0.00,0.36)	0.03 (0.00,1.17)	0.00 (0.00,0.36)
**6.23 (0.11,343.66)**	**H**	0.91 (0.16,5.31)	0.67 (0.03,17.37)	0.72 (0.00,118.58)	0.43 (0.01,27.86)	0.43 (0.15,1.21)	0.41 (0.08,2.02)	0.28 (0.01,8.35)	0.25 (0.01,7.37)	0.21 (0.01,4.11)	0.02 (0.00,1.48)
**6.84 (0.11,425.78)**	1.10 (0.19,6.40)	**I**	0.74 (0.02,22.11)	0.79 (0.00,143.21)	0.48 (0.01,34.37)	0.48 (0.11,1.99)	0.45 (0.07,2.94)	0.31 (0.01,10.57)	0.28 (0.01,9.34)	0.23 (0.01,5.30)	0.02 (0.00,1.82)
**9.28 (0.89,96.80)**	1.49 (0.06,38.50)	1.36 (0.05,40.69)	**F**	1.07 (0.02,54.85)	0.65 (0.00,103.58)	0.65 (0.03,14.12)	0.61 (0.02,16.76)	0.41 (0.15,1.12)	0.38 (0.16,0.90)	0.31 (0.02,4.49)	0.03 (0.00,1.77)
**8.71 (0.09,853.74)**	1.40 (0.01,231.36)	1.27 (0.01,231.93)	0.94 (0.02,48.27)	**L**	0.61 (0.00,374.73)	0.61 (0.00,90.30)	0.57 (0.00,98.30)	0.39 (0.01,22.65)	0.36 (0.01,20.10)	0.29 (0.00,34.00)	0.03 (0.00,8.34)
**14.38 (0.05,3862.88)**	2.31 (0.04,148.26)	2.10 (0.03,151.81)	1.55 (0.01,248.66)	1.65 (0.00,1022.08)	**D**	1.00 (0.02,56.46)	0.94 (0.01,63.60)	0.64 (0.00,113.54)	0.59 (0.00,101.40)	0.48 (0.00,65.16)	0.05 (0.00,15.64)
**14.38 (0.30,692.90)**	2.31 (0.82,6.46)	2.10 (0.50,8.79)	1.55 (0.07,33.89)	1.65 (0.01,246.29)	1.00 (0.02,56.46)	**C**	0.94 (0.28,3.19)	0.64 (0.03,16.43)	0.59 (0.02,14.47)	0.48 (0.03,7.90)	0.05 (0.00,3.00)
**15.31 (0.26,890.73)**	2.46 (0.50,12.16)	2.24 (0.34,14.70)	1.65 (0.06,45.58)	1.76 (0.01,303.83)	1.06 (0.02,72.07)	1.06 (0.31,3.62)	**E**	0.68 (0.02,21.86)	0.62 (0.02,19.31)	0.51 (0.02,10.86)	0.05 (0.00,3.81)
**22.37 (1.75,285.42)**	3.59 (0.12,107.63)	3.27 (0.09,113.02)	2.41 (0.89,6.50)	2.57 (0.04,149.54)	1.56 (0.01,274.89)	1.56 (0.06,39.77)	1.46 (0.05,46.70)	**G**	0.91 (0.24,3.41)	0.75 (0.04,12.93)	0.08 (0.00,4.83)
**24.50 (2.78,216.27)**	3.93 (0.14,113.94)	3.58 (0.11,119.81)	2.64 (1.11,6.29)	2.81 (0.05,159.18)	1.70 (0.01,294.41)	1.70 (0.07,42.02)	1.60 (0.05,49.47)	1.10 (0.29,4.10)	**J**	0.82 (0.05,13.60)	0.08 (0.00,5.14)
**29.95 (0.86,1048.54)**	4.80 (0.24,95.01)	4.38 (0.19,101.67)	3.23 (0.22,46.74)	3.44 (0.03,402.30)	2.08 (0.02,282.70)	2.08 (0.13,34.28)	1.96 (0.09,41.58)	1.34 (0.08,23.17)	1.22 (0.07,20.32)	**A**	0.10 (0.01,2.06)
**288.57 (2.77,30039.79)**	46.30 (0.68,3162.52)	42.18 (0.55,3232.98)	31.10 (0.56,1715.00)	33.15 (0.12,9165.72)	20.07 (0.06,6299.19)	20.07 (0.33,1206.72)	18.85 (0.26,1355.62)	12.90 (0.21,802.88)	11.78 (0.19,712.98)	9.64 (0.48,191.49)	**B**

Important results will be presented with a yellow background. A: LNG-IUS, B: LNG-IUS+medroxyprogesterone acetate, C: megestrol acetate, D: megestrol acetate+metformin, E: megestrol acetate+tamoxifen, F: medroxyprogesterone acetate, G: medroxyprogesterone acetate+metformin, H: STS inhibitor, I: general treatment, J: mTOR inhibitor, K: mTOR inhibitor+megestrol acetate+tamoxifen, L: tamoxifen.

#### 3.4.3 Other indicators

All p-values for comparisons between the studies were examined for both consistency and inconsistency, and the majority of p-values exceeded 0.05, signifying an acceptable level of consistency across studies (**Supplementary Tables 8**–**10**). The network maps illustrating the included interventions are presented in **Supplementary Figures 4A–C**.

##### 3.4.3.1 Pregnancy rate

**Figure 4:**
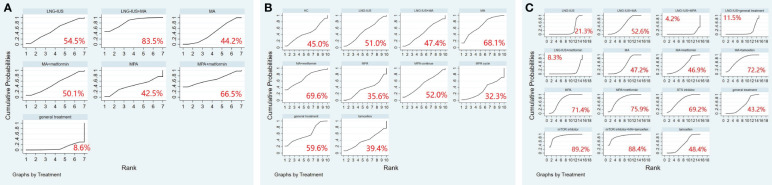
**(A)** SUCRA plot for pregnancy rate, **(B)** SUCRA plot for relapse rate, **(C)** SUCRA plot for adverse events. MA, megestrol acetate; MPA, medroxyprogesterone acetate; LNG-IUS, levonorgestrel-releasing intrauterine system; STS, steroid sulphatase; mTOR, mammalian target of rapamycin; HC, hydroxyprogesterone caproate.

A total of 9 randomized controlled trials and 7 interventions were included in the analysis of their effects on the pregnancy rate. The results of the network meta-analysis, with pregnancy rate as the outcome, indicated that both LNG-IUS+MA group [OR=9.05, 95% CI=(1.92, 42.62)] and MA group [OR=3.40, 95% CI=(1.17, 9.91)] were more effective than the general treatment group in enhancing the pregnancy rate (**Table 10**). In the SUCRA analysis, LNG-IUS+MA group ranked first (SUCRA=83.7%) in the probability ranking of different interventions for improving the pregnancy rate (**Figure 4A**).

**Table 10:** League table on pregnancy rate.

B	F	A	D	C	E	G
**B**	0.68 (0.02,27.31)	0.49 (0.18,1.32)	0.42 (0.09,2.05)	0.38 (0.12,1.15)	0.38 (0.01,14.03)	0.11 (0.02,0.52)
**1.48 (0.04,59.90)**	**F**	0.73 (0.02,30.33)	0.63 (0.02,25.35)	0.56 (0.02,18.97)	0.56 (0.26,1.21)	0.16 (0.00,6.54)
**2.04 (0.76,5.49)**	1.38 (0.03,57.61)	**A**	0.86 (0.17,4.46)	0.77 (0.23,2.57)	0.77 (0.02,29.45)	0.23 (0.04,1.13)
**2.36 (0.49,11.47)**	1.60 (0.04,64.60)	1.16 (0.22,5.99)	**D**	0.89 (0.29,2.70)	0.89 (0.02,33.09)	0.26 (0.06,1.22)
**2.66 (0.87,8.17)**	1.80 (0.05,61.29)	1.30 (0.39,4.37)	1.13 (0.37,3.43)	**C**	1.00 (0.03,31.26)	0.29 (0.10,0.86)
**2.66 (0.07,99.41)**	1.80 (0.83,3.91)	1.30 (0.03,50.10)	1.13 (0.03,41.95)	1.00 (0.03,31.26)	**E**	0.29 (0.01,10.81)
**9.05 (1.92,42.62)**	6.11 (0.15,244.21)	4.43 (0.88,22.28)	3.83 (0.82,17.92)	3.40 (1.17,9.91)	3.40 (0.09,125.05)	**G**

Important results will be presented with a yellow background. A: LNG-IUS, B: LNG-IUS+megestrol acetate, C: megestrol acetate, D: megestrol acetate+metformin, E: medroxyprogesterone acetate, F: medroxyprogesterone acetate+metformin, G: general treatment.

##### 3.4.3.2 Relapse rate

A total of 10 randomized controlled trials and 10 interventions underwent analysis to assess their impact on relapse rate. The results of the network meta-analysis, considering relapse rate as the outcome, revealed that the general treatment group [OR=1.38, 95% CI=(1.02, 1.85)] caused more relapses compared to the MPA group (**Table 11**). In the SUCRA analysis, LNG-IUS+MA group ranked last (SUCRA=47.4%) in the probability ranking of different interventions for increasing the relapse rate among all combined treatment regimens (**Figure 4B**).

**Table 11:** League table on relapse rate.

E	D	I	G	B	C	A	J	F	H
**E**	0.89 (0.28,2.79)	0.52 (0.01,47.93)	0.58 (0.09,3.62)	0.58 (0.11,3.01)	0.56 (0.13,2.48)	0.44 (0.00,40.73)	0.39 (0.00,37.12)	0.38 (0.00,34.49)	0.38 (0.06,2.45)
**1.13** **(0.36,3.55)**	**D**	0.59 (0.01,46.67)	0.66 (0.16,2.73)	0.65 (0.20,2.13)	0.63 (0.25,1.63)	0.49 (0.01,39.68)	0.43 (0.01,36.21)	0.43 (0.01,33.58)	0.43 (0.10,1.86)
**1.91 (0.02,175.61)**	1.70 (0.02,134.15)	**I**	1.11 (0.01,110.35)	1.11 (0.01,102.59)	1.08 (0.01,94.11)	0.84 (0.59,1.19)	0.74 (0.35,1.54)	0.73 (0.54,0.98)	0.72 (0.01,72.83)
**1.72 (0.28,10.75)**	1.53 (0.37,6.36)	0.90 (0.01,89.35)	**G**	1.00 (0.45,2.21)	0.97 (0.21,4.39)	0.75 (0.01,75.92)	0.66 (0.01,69.15)	0.65 (0.01,64.32)	0.65 (0.26,1.61)
**1.73** **(0.33,8.99)**	1.53 (0.47,5.00)	0.90 (0.01,83.63)	1.00 (0.45,2.23)	**B**	0.97 (0.27,3.51)	0.76 (0.01,71.07)	0.67 (0.01,64.77)	0.66 (0.01,60.19)	0.65 (0.27,1.57)
**1.78** **(0.40,7.86)**	1.58 (0.61,4.05)	0.93 (0.01,81.36)	1.03 (0.23,4.69)	1.03 (0.28,3.72)	**C**	0.78 (0.01,69.15)	0.69 (0.01,63.05)	0.68 (0.01,58.55)	0.67 (0.14,3.19)
**2.28 (0.02,212.41)**	2.02 (0.03,162.34)	1.19 (0.84,1.70)	1.33 (0.01,133.44)	1.32 (0.01,124.09)	1.28 (0.01,113.86)	**A**	0.88 (0.39,1.99)	0.87 (0.55,1.37)	0.86 (0.01,88.07)
**2.60 (0.03,250.36)**	2.30 (0.03,191.57)	1.36 (0.65,2.83)	1.51 (0.01,157.19)	1.50 (0.02,146.24)	1.46 (0.02,134.24)	1.14 (0.50,2.57)	**J**	0.99 (0.47,2.05)	0.98 (0.01,103.72)
**2.63 (0.03,239.33)**	2.33 (0.03,182.77)	1.38 (1.02,1.85)	1.53 (0.02,150.41)	1.52 (0.02,139.82)	1.48 (0.02,128.25)	1.15 (0.73,1.83)	1.01 (0.49,2.11)	**F**	0.99 (0.01,99.27)
**2.65** **(0.41,17.17)**	2.35 (0.54,10.27)	1.38 (0.01,139.58)	1.54 (0.62,3.81)	1.53 (0.64,3.70)	1.49 (0.31,7.07)	1.16 (0.01,118.58)	1.02 (0.01,108.01)	1.01 (0.01,100.48)	**H**

Important results will be presented with a yellow background. A: hydroxyprogesterone caproate, B: LNG-IUS, C: LNG-IUS+megestrol acetate, D: megestrol acetate, E: megestrol acetate+metformin, F: medroxyprogesterone acetate, G: medroxyprogesterone acetate continue, H: medroxyprogesterone acetate cycle, I: general treatment, J: tamoxifen.

##### 3.4.3.3 Adverse events

We included 15 randomized controlled trials and 15 interventions to analyze their safety. The results of the network meta-analysis showed that more adverse events occurred in mTOR inhibitor group [OR=4.38, 95% CI=(1.34, 7.42)], mTOR+ MA+tamoxifen group [OR=4.41, 95% CI=(1.10, 7.73)], and MPA group [OR=2.94, 95% CI=(1.03, 4.85)] compared to LNG-IUS+MA group. Similarly, more adverse events occurred in mTOR inhibitor group [OR=4.59, 95% CI=(1.62, 7.57)], mTOR+ MA+tamoxifen group [OR=4.63, 95% CI=(1.38, 7.88)], MPA+metformin group [OR=3.40, 95% CI=(1.27, 5.53)], MA+tamoxifen group [OR=3.22, 95% CI=(1.16, 5.27)], MPA group [OR=3.16, 95% CI=(1.36, 4.96)], and STS inhibitor group [OR=3.07, 95% CI=(1.09, 5.06)] compared to LNG-IUS+ metformin group (**Supplementary Table 11**). In the SUCRA analysis, Among all treatment regimens, LNG-IUS+MPA group ranked last (SUCRA=4.2%) in the probability ranking of the incidence of adverse events due to the different interventions and correspondingly, mTOR inhibitor group and mTOR inbitor+MA+tamoxifen group were ranked first (SUCRA=89.2%) and second (SUCRA=88.4%) in the probability ranking, respectively (**Figure 4C**).

### 3.5 Publication bias test

We generated distinct funnel plots for each outcome indicator to identify potential publication bias. Upon careful examination of the funnel plots, no significant publication bias was observed. Detailed information is presented in **Supplementary Figure 5**.

## 4 Discussion

In this investigation, we conducted a comprehensive comparison of the effectiveness of various progestin-based regimens for treating patients with endometrial cancer or endometrial atypia. A total of 5,323 patients diagnosed with endometrial cancer or atypical hyperplasia, sourced from 27 studies, were included, encompassing 19 different interventions. Among these interventions, 9 comprised progestin-based combination therapy programs. Our findings indicated that the Hydroxyprogesterone caproate (HC)+tamoxifen regimen demonstrated superior efficacy in extending overall survival (OS), while the mTOR inhibitor+megestrol acetate (MA)+tamoxifen regimen exhibited the best performance in prolonging progression-free survival (PFS). Regarding efficacy indicators, the LNG-IUS+medroxyprogesterone acetate (MPA) regimen emerged as the top choice for increasing the number of complete responses (CR). The MA+tamoxifen regimen proved to be the optimal treatment for enhancing the number of partial responses (PR). For the objective response rate (ORR), the LNG-IUS+MPA regimen stood out as the most effective combination therapy. In terms of stable disease (SD), the mTOR inhibitor+MA+tamoxifen regimen demonstrated superior efficacy. In the context of disease progression, the LNG-IUS+MPA regimen exhibited the lowest likelihood of causing disease progression. Additionally, the LNG-IUS+MA regimen emerged as the preferred combination therapy for improving pregnancy rates, and it was also the least likely to result in disease relapse. However, in terms of safety, mTOR inhibitor and mTOR inhibitor+MA+tamoxifen had the highest likelihood of adverse events, while LNG-IUS+MPA was the safest combination regimen. Overall, combination therapy based on mTOR inhibitors had advantages in improving certain indicators, but it also faced the risk of adverse events. The combination therapy based on tamoxifen had a lower risk of adverse events compared to mTOR inhibitor combination therapy, but the indicators it improves were limited. The LNG-IUS-based dual progestin regimen not only had an advantage in the number of indicators improved but was also the safest approach. Therefore, among all the combination regimens, the LNG-IUS-based dual progestin regimen may represent the most suitable approach for treating endometrial cancer or atypical endometrial hyperplasia.

In our investigation, tamoxifen featured prominently in optimal combination regimens associated with prolonged survival index, and it was also a key component in the optimal combination regimen employing PR and SD as outcome indicators. Existing studies have highlighted that estrogen replacement therapy without progestin supplementation leads to a substantial increase (4- to 14-fold) in the relative risk of endometrial cancer (48). Tamoxifen, classified as a selective estrogen receptor modulator (SERM), functions by obstructing estrogen binding to estrogen receptors in endometrial cancer (44, 49). Consequently, it holds potential beneficial effects in managing endometrial cancer or atypical endometrial hyperplasia. Hald et al. investigated tamoxifen’s efficacy in advanced endometrial cancer, reporting assessable disease remission (partial and complete) in 8 of 33 patients (50). Quinn et al. observed a 20% remission rate in 10 out of 49 patients with advanced/recurrent endometrial cancer treated with tamoxifen (51). However, the relationship between tamoxifen and an elevated risk of endometrial cancer is a subject of extensive discourse. Leeuwen et al. conducted a case-control study in the Netherlands, revealing a trend toward a significantly increased risk of endometrial cancer with longer duration of tamoxifen use and an escalating cumulative dose (52). In conclusion, the role of tamoxifen in endometrial cancer, along with its specific mechanisms, warrants further exploration.

High-frequency aberrations on the PI3K/Akt/mTOR pathway have been identified in endometrial cancer (31). Elevated activity of the PI3K/Akt/mTOR pathway is linked to resistance to progesterone treatment in endometrial cancer cell lines and mouse models, and inhibition of this pathway can reverse such resistance (53). Consequently, mTOR inhibitors demonstrate promise as targeted agents for endometrial cancer treatment. In our investigation, mTOR inhibitors emerged as crucial components in the optimal combination regimen for achieving positive outcomes in terms of progression-free survival (PFS) and stable disease (SD). First-generation mTOR inhibitors currently undergoing clinical trials for advanced or recurrent endometrial cancer include everolimus (RA001), temsirolimus (CCI779), and ridaforolimus (AP2357) (54). A phase II clinical trial of ridaforolimus (AP2357) in patients with advanced and recurrent endometrial cancer revealed a clinical benefit response (CBR) in 9 out of 27 patients with evaluable responses (55). Tsoref et al. conducted a phase II trial assessing the tolerability and activity of oral ridaforolimus (AP2357) in patients with metastatic or recurrent endometrial cancer, reporting that among the 31 evaluable patients, 3 achieved partial response (PR), and an additional 18 achieved stable disease (SD) (56). The application of mTOR inhibitors restrains the proliferation and progression of cancer cells, and when combined with progestins, they may exert synergistic effects.

Among the various optimal combination regimens, the LNG-IUS-based dual progestin regimen emerged prominently, underscoring the crucial role of LNG-IUS in treating endometrial cancer or atypical endometrial hyperplasia. LNG-IUS serves as a long-acting contraceptive option for women and is employed in the management of abnormal and heavy bleeding (heavy periods). Its mechanism involves the inhibition of endometrial proliferation, leading to endometrial atrophy through metaplasia and suppression of endometrial glands (57). This effect may stem from LNG-IUS inhibiting the synthesis of endometrial estrogen receptors, rendering the endometrium insensitive to circulating estrogen. Furthermore, the therapeutic efficacy relies significantly on local progestogen action within the uterine cavity, potentially making it preferable to other progestins (58). LNG-IUS demonstrates heightened effectiveness in reversing endometrial hyperplasia. For example, Varma et al. conducted a prospective study involving 105 women diagnosed with endometrial hyperplasia treated with LNG-IUS, revealing a remarkable 90% regression within 2 years, with 92% resolution of atypical endometrial hyperplasia (59). In another study, Hashim et al. conducted a randomized controlled trial involving 120 perimenopausal women with atypical endometrial hyperplasia, noting a significantly higher regression rate in the LNG-IUS group compared to the oral progestogen group, achieving regression within 1 year (60). Additionally, LNG-IUS plays a pivotal role in endometrial cancer treatment. Westin et al. conducted a phase II trial of LNG-IUS in 57 patients diagnosed with complicated atypical hyperplasia and early endometrial cancer, reporting an overall 83% 12-month remission rate (RR), with a 66.7% remission rate for grade 1 endometrial-looking endometrial cancer (61). However, it’s worth noting that LNG-IUS may face challenges in delivering a uniform progesterone dose to the entire endometrial cavity, potentially creating a ‘myometrial sanctuary’ for tumors (62).

In terms of progestin therapy for EC or AEH, relevant guidelines are also described, for example, for patients with grade 1, stage IA endometrial cancer who wish to preserve fertility, both the National Comprehensive Cancer Network (NCCN) and European guidelines recommend continuous progestin-based therapy, which includes MA, MPA, or LNG-IUS. In addition, for patients with disseminated metastases, who are unsuitable for local therapy, or who have further recurrence, both the NCCN and the European guidelines recommend hormonal therapy, with the European Society of Gynaecological Oncology (ESGO) guidelines recommending the use of MA and MPA (63). Treatment guidelines recommend that patients with atypical endometrial hyperplasia should be treated with hysterectomy. However there is still controversy over the use of progestins (64). Although some guidelines do not make a clear statement about progestin-based combination therapy regimens, this is the innovation we wanted to explore. The results of a meta-analysis showed that oral progestins still resulted in high CR rates and relapse rates within the accepted range for patients with AEH or EC who relapsed after receiving conservative treatment (65). Through this study, we want to find an efficacious and safe progestin-based combination therapy regimen, to raise awareness of progestin therapy for endometrial cancer, and to lay the foundation for subsequent research.

Notably, alterations occurring in the endometrium can link reproductive and oncological diseases. It has been shown that polycystic ovary syndrome (PCOS), a heterogeneous endocrine disease, causes dysregulation of endometrial sex hormone receptor and co-receptor expression, endometrial insulin resistance, impaired glucose transport and utilization, chronic inflammation, immune dysfunction, uterine vascular alterations, and even abnormal gene expression and cellular abnormalities of the endometrium, which make the endometrium dysfunctional (66). A national case-control study from Australia showed a four-fold increased risk of endometrial cancer in patients with PCOS compared to those without PCOS, and in addition, hirsutism and extremely irregular menstruation among the symptoms of PCOS were significantly associated with the risk of endometrial cancer (67). Results from another cohort study from Denmark showed a similar fourfold increase in endometrial risk (68). This is consistent with the results of a number of meta-analyses of which we are aware. For example a meta-analysis that included 10 studies showed that women with PCOS had a significantly increased risk of endometrial cancer compared to controls (69), with a further increase in risk when postmenopausal people were excluded from the meta-analysis. Similarly, adenomyosis is a benign gynaecological disease, whereas EC can originate from malignant transformation of ectopic endometrium within the adenomyosis lesion, leading to endometrial carcinoma arising in adenomyosis (EC-AIA). This disease is difficult to diagnose preoperatively, but differs from EC in postoperative histological features and prognosis (70). We therefore envisage that the combination of hysteroscopic surgery and progestins will yield beneficial results in the treatment of endometrial carcinoma and its rarer types.

Previous risk stratification of EC has been based on pathological features, and studies of The Cancer Genome Atlas (TCGA) may have changed our view. In TCGA, EC can be classified into four prognostically relevant groups based on mutation load and somatic copy number variation, namely POLE-mutant, mismatch repair (MMR)-deficient, p53-abnormal, and “no specific molecular profile” (NSMP). Each group differs in pathological characteristics, prognosis, and response to different therapies, making the TGCA classification of great advantage for risk stratification and management of EC (71).

This study has certain advantages. Firstly, our study investigates the potential of progestin-based combination therapy regimens in treating endometrial cancer or atypical endometrial hyperplasia and finds the combination regimen with the best efficacy and the least number of adverse events, offering significant clinical implications. Secondly, the rigorous application of the network meta-analysis method ensures a high level of medical evidence, delivering crucial information for clinicians and policymakers. We also assessed the risk of bias for each of the included studies, and none found the presence of serious bias. Finally, the inclusion of randomized controlled trials and a rigorous data extraction process ensure the quality of the study. However, our study has some limitations. While we incorporated 27 studies and analyzed data from 5323 patients, the persuasiveness of our findings could be further strengthened with a more extensive literature review. Additionally, we acknowledge a lack of in-depth consideration of heterogeneity among the studies. Factors like patient age, weight, progesterone dosage, and administration route were not thoroughly addressed and may introduce confounding variables. For the EC and AEH patients involved in this study, we did not analyze them separately. Finally, although we chose multiple indicators to assess, the number of studies included in each indicator was not the same, which may have had some impact on the results.

In summary, our study indicates that while tamoxifen and mTOR inhibitors show efficacy in combined progestin-based regimens, a dual progestin-based approach utilizing LNG-IUS has better efficacy and safety and holds greater promise for treating endometrial cancer or endometrial atypical hyperplasia. This insight could be valuable for clinicians in selecting the most suitable progestin treatment for patients with endometrial cancer or atypical endometrial hyperplasia.

## Data availability statement

The original contributions presented in the study are included in the article/**Supplementary Material**. Further inquiries can be directed to the corresponding author.

## Author contributions

JC: Conceptualization, Methodology, Writing – original draft. YZ: Data curation, Writing – review & editing. LS: Writing – review & editing. T-JW: Conceptualization, Writing – review & editing.
