# Supplementary material for: Comparative effects of progestin-based combination therapy for endometrial cancer or atypical endometrial hyperplasia: a systematic review and network meta-analysis
Source: Front Oncol. 2024 May 3;14:1391546. doi: 10.3389/fonc.2024.1391546 (PMC11099254; doi:10.3389/fonc.2024.1391546)
Supplement: Supplementary file 1 [file DataSheet_1.docx]

Supplementary Material

# Supplementary Data

**Supplementary Table 1.** Consistency test for overall survival (OS).

**Supplementary Table 2.** Consistency test for progression-free survival (PFS).

**Supplementary Table 3.** Consistency test for complete response (CR).

**Supplementary Table 4.** Consistency test for partial response (PR).

**Supplementary Table 5.** Consistency test for objective response rate (ORR).

**Supplementary Table 6.** Consistency test for stable disease (SD).

**Supplementary Table 7.** Consistency test for progressive disease (PD).

**Supplementary Table 8.** Consistency test for pregnancy rate.

**Supplementary Table 9.** Consistency test for relapse rate.

**Supplementary Table 10.** Consistency test for adverse events.

**Supplementary Table 11.** League table on adverse events.

**Supplementary Figure 1.** (**A**) Risk of bias of the included studies, (**B**) Overall risk of bias.

**Supplementary Figure 2.** (**A**) Network map for overall survival (OS), (**B**) Network map for progression-free survival (PFS). HC: hydroxyprogesterone caproate, MA: megestrol acetate, MPA: medroxyprogesterone acetate, STS: steroid sulphatase, mTOR: mammalian target of rapamycin.

**Supplementary Figure 3.** (**A**) Network map for complete response (CR), (**B**) Network map for partial response (PR), (**C**) Network map for objective response rate (ORR), (**D**) Network map for stable disease (SD), (**E**) Network map for progressive disease (PD). MA: megestrol acetate, MPA: medroxyprogesterone acetate, STS: steroid sulphatase, mTOR: mammalian target of rapamycin. LNG-IUS: levonorgestrel-releasing intrauterine system.

**Supplementary Figure 4.** (**A**) Network map for pregnancy rate, (**B**) Network map for relapse rate. (**C**) Network map for adverse events. MA: megestrol acetate, MPA: medroxyprogesterone acetate, LNG-IUS: levonorgestrel-releasing intrauterine system. HC: hydroxyprogesterone caproate. STS: steroid sulphatase, mTOR: mammalian target of rapamycin.

**Supplementary Figure 5.** Funnel plot on publication bias. (**A**) OS, (**B**) PFS, (**C**) CR, (**D**) PR, (**E**) ORR, (**F**) SD, (**G**) PD, (**H**) Pregnancy rate, (**I**) Relapse rate, (J) Adverse events.

# Supplementary Figures and Tables

**Supplementary Table 1.** Consistency test for overall survival (OS).

| Coef. Std. Err. z P>\|z\| [95% Conf. Interval] |
| --- |
| B VS CON 1.831322 2.033754 0.90 0.368 -2.154762 5.817406 |
| C VS CON 2.128569 2.327817 0.91 0.361 -2.433869 6.691007 |
| D VS CON 1.098181 1.790259 0.61 0.540 -2.410662 4.607025 |
| E VS CON .482993 2.056535 0.23 0.814 -3.547742 4.513728 |
| F VS CON .9214769 2.01566 0.46 0.648 -3.029143 4.872097 |
| G VS CON .9923969 1.975081 0.50 0.615 -2.87869 4.863484 |
| H VS CON -.6879432 2.641656 0.26 0.795 -5.865494 4.489608 |
| I VS CON -1.09732 2.795983 0.39 0.695 -6.577347 4.382706 |
| J VS CON 1.179618 2.088528 0.56 0.572 -2.913822 5.273058 |

Con: STS inhibitor, B: hydroxyprogesterone caproate, C: hydroxyprogesterone caproate+tamoxifen, D: megestrol acetate, E: megestrol acetate+tamoxifen, F: medroxyprogesterone acetate, G: general treatment, H: mTOR inhibitor, I: mTOR inhibitor+megestrol acetate+tamoxifen, J: tamoxifen.

**Supplementary Table 2.** Consistency test for progression-free survival (PFS).

| Coef. Std. Err. z P>\|z\| [95% Conf. Interval] |
| --- |
| B VS CON .1997894 .4384068 0.46 0.649 -.6594721 1.059051 |
| C VS CON -.0285734 2.014238 -0.01 0.989 -3.976407 3.91926 |
| D VS CON -.165988 .4078268 -0.41 0.684 -.9653138 .6333379 |
| E VS CON 1.298367 1.7032 0.76 0.446 -2.039843 4.636576 |
| F VS CON 1.489422 1.930093 0.77 0.440 -2.293491 5.272335 |

Con: megestrol acetate, B: medroxyprogesterone acetate, C: STS inhibitor, D: general treatment, E: mTOR inhibitor, F: mTOR inhibitor+megestrol acetate+tamoxifen.

**Supplementary Table 3.** Consistency test for complete response (CR).

| Coef. Std. Err. z P>\|z\| [95% Conf. Interval] |
| --- |
| B VS CON -.2172958 .4652883 -0.47 0.640 -1.129244 .6946525 |
| C VS CON 1.518924 .4858941 3.13 0.002 .5665889 2.471259 |
| D VS CON .2623643 .5131147 0.51 0.609 -.7433221 1.268051 |
| E VS CON -.1431008 .4734399 -0.30 0.762 -1.071026 .7848242 |
| F VS CON -.3366632 .4480889 -0.75 0.452 -1.214901 .541575 |
| G VS CON .1478448 .5755507 0.26 0.797 -.9802138 1.275903 |
| H VS CON -1.105796 1.50924 -0.73 0.464 -4.063852 1.85226 |
| I VS CON -1.172637 .3465042 -3.38 0.001 -1.851773 -.4935016 |
| J VS CON -1.749667 1.562113 -1.12 0.263 -4.811352 1.312018 |
| K VS CON -3.878877 1.451446 -2.67 0.008 -6.723658 -1.034096 |
| L VS CON -.5142213 .4805254 -1.07 0.285 -1.456034 .4275912 |
| M VS CON -1.973881 1.629831 -1.21 0.226 -5.168291 1.22053 |
| N VS CON -.7226155 .5747452 -1.26 0.209 -1.849095 .4038643 |
| O VS CON -2.313298 1.681897 -1.38 0.169 -5.609755 .9831582 |
| P VS CON -3.466532 2.276413 -1.52 0.128 -7.928219 .995155 |
| Q VS CON -1.435002 .67998 -2.11 0.035 -2.767738 -.1022652 |

Con: LNG-IUS, B: LNG-IUS+megestrol acetate, C: LNG-IUS+medroxyprogesterone acetate, D: LNG-IUS+general treatment, E: LNG-IUS+metformin, F: megestrol acetate, G: megestrol acetate+metformin, H: megestrol acetate+tamoxifen, I: medroxyprogesterone acetate, J: medroxyprogesterone acetate continue, K: medroxyprogesterone acetate cycle, L: medroxyprogesterone acetate+metformin, M: STS inhibitor, N: general treatment, O: mTOR inhibitor, P: mTOR inhibitor+megestrol acetate+tamoxifen, Q: tamoxifen.

**Supplementary Table 4.** Consistency test for partial response (PR).

| Coef. Std. Err. z P>\|z\| [95% Conf. Interval] |
| --- |
| B VS CON -.7308875 .6788418 -1.08 0.282 -2.061393 .599618 |
| C VS CON 1.040245 1.682444 0.62 0.536 -2.257286 4.337775 |
| D VS CON -.8374572 2.345658 -0.36 0.721 -5.434863 3.759948 |
| E VS CON 1.165408 1.842337 0.63 0.527 -2.445506 4.776321 |
| F VS CON .2308866 1.668879 0.14 0.890 -3.040056 3.501829 |
| G VS CON .6273139 1.702465 0.37 0.713 -2.709456 3.964084 |
| H VS CON -.3643988 1.825183 -0.20 0.842 -3.941692 3.212894 |
| I VS CON .6993181 1.722742 0.41 0.685 -2.677195 4.075831 |
| J VS CON -.9098125 2.343869 -0.39 0.698 -5.503712 3.684087 |
| K VS CON -1.043344 2.455895 -0.42 0.671 -5.85681 3.770122 |
| L VS CON -.1345731 1.736251 -0.08 0.938 -3.537563 3.268417 |

Con: LNG-IUS, B: LNG-IUS+medroxyprogesterone acetate, C: megestrol acetate, D: megestrol acetate+metformin, E: megestrol acetate+tamoxifen, F: medroxyprogesterone acetate, G: medroxyprogesterone acetate+metformin, H: STS inhibitor, I: general treatment, J:mTOR inhibitor, K: mTOR inhibitor+megestrol acetate+tamoxifen, L: tamoxifen.

**Supplementary Table 5.** Consistency test for objective response rate (ORR).

| Coef. Std. Err. z P>\|z\| [95% Conf. Interval] | | | | | | |
| --- | --- | --- | --- | --- | --- | --- |
| B VS CON | **-.0502567** | **.5739763** | **-0.09** | **0.930** | **-1.175229** | **1.074716** |
| C VS CON | **-.267567** | **.5148304** | **-0.52** | **0.603** | **-1.276616** | **.741482** |
| D VS CON | **1.651646** | **.8403216** | **1.97** | **0.049** | **.0046464** | **3.298647** |
| E VS CON | **.2121076** | **.7698932** | **0.28** | **0.783** | **-1.296855** | **1.72107** |
| F VS CON | **-.1933575** | **.744039** | **-0.26** | **0.795** | **-1.651647** | **1.264932** |
| G VS CON | **-.3869409** | **.3587106** | **-1.08** | **0.281** | **-1.090001** | **.3161189** |
| H VS CON | **-.4475655** | **.7717416** | **-0.58** | **0.562** | **-1.960151** | **1.06502** |
| I VS CON | **-1.222893** | **.659604** | **-1.85** | **0.064** | **-2.515693** | **.0699072** |
| J VS CON | **-1.799924** | **1.664225** | **-1.08** | **0.279** | **-5.061746** | **1.461898** |
| K VS CON | **-3.929134** | **1.560815** | **-2.52** | **0.012** | **-6.988275** | **-.8699922** |
| L VS CON | **-.2404394** | **.7440701** | **-0.32** | **0.747** | **-1.69879** | **1.217911** |
| M VS CON | **-2.050867** | **.7846292** | **- 2.61** | **0.009** | **-3.588712** | **-.513022** |
| N VS CON | **-1.307872** | **.5539055** | **-2.36** | **0.018** | **-2.393507** | **-.2222372** |
| O VS CON | **-2.896111** | **1.696347** | **-1.71** | **0.088** | **-6.220891** | **.4286677** |
| P VS CON | **-3.422205** | **1.839302** | **-1.86** | **0.063** | **-7.02717** | **.1827614** |
| Q VS CON | **-1.650546** | **.7848415** | **-2.10** | **0.035** | **-3.188807** | **-.1122846** |

Con: megestrol acetate+metformin, B: LNG-IUS, C: LNG-IUS+megestrol acetate, D: LNG-IUS+medroxyprogesterone acetate, E: LNG-IUS+general treatment,F: LNG-IUS+metformin, G: megestrol acetate, H: megestrol acetate+tamoxifen, I: medroxyprogesterone acetate, J: medroxyprogesterone acetate continue,K: medroxyprogesterone acetate cycle, L: medroxyprogesterone acetate+metformin, M: STS inhibitor, N: general treatment, O: mTOR inhibitor, P: mTOR inhibitor+megestrol acetate+tamoxifen, Q: tamoxifen.

**Supplementary Table 6.** Consistency test for stable disease (SD).

| Coef. Std. Err. z P>\|z\| [95% Conf. Interval] |
| --- |
| B VS CON -1.474224 .8547892 -1.72 0.085 -3.149581 .2011317 |
| C VS CON .4857311 1.341108 0.36 0.717 -2.142792 3.114254 |
| D VS CON -.612882 1.818531 -0.34 0.736 -4.177137 2.951373 |
| E VS CON .879074 2.209057 0.40 0.691 -3.450598 5.208746 |
| F VS CON .5686552 .9233736 0.62 0.538 -1.241124 2.378434 |
| G VS CON -.2882809 1.149892 -0.25 0.802 -2.542028 1.965466 |
| H VS CON 1.108474 1.541161 0.72 0.472 -1.912147 4.129094 |
| I VS CON 1.330108 1.538129 0.86 0.387 -1.684569 4.344786 |
| J VS CON 1.551991 1.198399 1.30 0.195 -.7968271 3.90081 |
| K VS CON 1.567256 1.43144 1.09 0.274 -1.238315 4.372827 |
| L VS CON .9963071 1.17275 0.85 0.396 -1.302241 3.294855 |

Con: LNG-IUS, B: LNG-IUS+medroxyprogesterone acetate, C: megestrol acetate, D: megestrol acetate+metformin, E: megestrol acetate+tamoxifen, F: medroxyprogesterone acetate, G: medroxyprogesterone acetate+metformin, H: STS inhibitor, I: general treatment, J: mTOR inhibitor, K: mTOR inhibitor+megestrol acetate+tamoxifen, L: tamoxifen.

**Supplementary Table 7.** Consistency test for progressive disease (PD).

| Coef. Std. Err. z P>\|z\| [95% Conf. Interval] |
| --- |
| B VS CON -2.265433 1.525234 -1.49 0.137 -5.254836 .7239702 |
| C VS CON .7336927 1.428916 0.51 0.608 -2.066931 3.534316 |
| D VS CON .7336927 2.505413 0.29 0.770 -4.176827 5.644212 |
| E VS CON .6711723 1.55937 0.43 0.667 -2.385137 3.727481 |
| F VS CON 1.171523 1.363689 0.86 0.390 -1.501258 3.844304 |
| G VS CON .2916027 1.454644 0.20 0.841 -2.559447 3.142652 |
| H VS CON 1.569562 1.522553 1.03 0.303 -1.414587 4.55371 |
| I VS CON 1.476437 1.604643 0.92 0.358 -1.668606 4.621479 |
| J VS CON .2007441 1.433947 0.14 0.889 -2.609741 3.011229 |
| K VS CON 3.399417 1.814083 1.87 0.061 -.1561211 6.954956 |
| L VS CON 1.235375 2.429453 0.51 0.611 -3.526266 5.997016 |

Con: LNG-IUS, B: LNG-IUS+medroxyprogesterone acetate, C: megestrol acetate, D: megestrol acetate+metformin, E: megestrol acetate+tamoxifen, F: medroxyprogesterone acetate, G: medroxyprogesterone acetate+metformin, H: STS inhibitor, I: general treatment, J: mTOR inhibitor, K: mTOR inhibitor+megestrol acetate+tamoxifen, L: tamoxifen.

**Supplementary Table 8.** Consistency test for pregnancy rate.

| Coef. Std. Err. z P>\|z\| [95% Conf. Interval] |
| --- |
| B VS CON .7134855 .5050463 1.41 0.158 -.276387 1.703358 |
| C VS CON -.265578 .6166514 -0.43 0.667 -1.474192 .9430365 |
| D VS CON -.1469721 .8382083 -0.18 0.861 -1.78983 1.495886 |
| E VS CON -.2655615 1.861452 -0.14 0.887 -3.91394 3.382817 |
| F VS CON .3208327 1.904578 0.17 0.866 -3.412072 4.053737 |
| G VS CON -1.48952 .8234862 -1.81 0.070 -3.103524 .1244829 |

Con: LNG-IUS, B: LNG-IUS+megestrol acetate, C: megestrol acetate, D: megestrol acetate+metformin, E: medroxyprogesterone acetate, F: medroxyprogesterone acetate+metformin, G: general treatment.

**Supplementary Table 9.** Consistency test for relapse rate.

| Coef. Std. Err. z P>\|z\| [95% Conf. Interval] |
| --- |
| B VS CON .2786814 2.317547 0.12 0.904 -4.263627 4.82099 |
| C VS CON .2493084 2.288626 0.11 0.913 -4.236316 4.734933 |
| D VS CON .7044483 2.237425 0.31 0.753 -3.680824 5.089721 |
| E VS CON .8258091 2.312659 0.36 0.721 -3.706919 5.358537 |
| F VS CON -.1427261 .2349335 -0.61 0.544 -.6031873 .3177351 |
| G VS CON .2820203 2.352934 0.12 0.905 -4.329645 4.893686 |
| H VS CON -.1487626 2.360685 -0.06 0.950 -4.775621 4.478096 |
| I VS CON .1765106 .1803031 0.98 0.328 -.1768769 .5298982 |
| J VS CON -.128529 .4164037 -0.31 0.758 -.9446652 .6876073 |

Con: hydroxyprogesterone caproate, B: LNG-IUS, C: LNG-IUS+megestrol acetate, D: megestrol acetate, E: megestrol acetate+metformin, F: medroxyprogesterone acetate, G: medroxyprogesterone acetate continue, H: medroxyprogesterone acetate cycle, I: general treatment, J: tamoxifen.

**Supplementary Table 10.** Consistency test for adverse events.

| Coef. Std. Err. z P>\|z\| [95% Conf. Interval] |
| --- |
| B VS CON 1.524677 .5417804 2.81 0.005 .4628065 2.586547 |
| C VS CON -1.391208 .6583335 -2.11 0.035 -2.681518 -.1008984 |
| D VS CON -.7252355 .667132 -1.09 0.277 -2.03279 .5823192 |
| E VS CON -.9420433 .5827914 -1.62 0.106 -2.084293 .2002069 |
| F VS CON 1.382656 .5283216 2.62 0.009 .3471651 2.418148 |
| G VS CON 1.314546 .7653926 1.72 0.086 -.1855959 2.814688 |
| H VS CON 2.273628 .8731399 2.60 0.009 .5623053 3.984951 |
| I VS CON 2.216833 .7113635 3.12 0.002 .8225858 3.611079 |
| J VS CON 2.459298 .9177371 2.68 0.007 .6605667 4.25803 |
| K VS CON 2.131892 .8294312 2.57 0.010 .5062366 3.757547 |
| L VS CON 1.250921 .8503386 0.141 0.141 -.4157121 2.917554 |
| M VS CON 3.652669 1.401083 2.61 0.091 .9065957 6.398742 |
| N VS CON 3.687757 1.553555 2.37 0.181 .6428452 6.732668 |
| O VS CON 1.431964 .815055 1.76 0.079 -.1655147 3.029442 |

Con: LNG-IUS, B: LNG-IUS+ megestrol acetate, C: LNG-IUS+ medroxyprogesterone acetate, D: LNG-IUS+ general treatment, E: LNG-IUS+ metformin, F: megestrol acetate, G: megestrol acetate +metformin, H: megestrol acetate +tamoxifen, I: medroxyprogesterone acetate, J: medroxyprogesterone acetate+metformin, K:STS inhibitor, L:general treatment, M:mTOR inhibitor, N:mTOR+ megestrol acetate+tamoxifen, O:tamoxifen.

| M | N | J | H | I | K | B | O | F | G | L | A | D | E | C |
| --- | --- | --- | --- | --- | --- | --- | --- | --- | --- | --- | --- | --- | --- | --- |
| M | 0.04 (-1.28,1.35) | -1.19 (-3.82,1.43) | -1.38 (-4.57,1.81) | -1.44 (-3.80,0.93) | -1.52 (-4.66,1.62) | -2.13 (-5.04,0.78) | -2.22 (-4.71,0.27) | -2.27 (-5.15,0.61) | -2.34 (-5.42,0.74) | -2.40 (-4.94,0.13) | -3.65 (-6.40,-0.91) | -4.38 (-7.42,-1.34) | -4.59 (-7.57,-1.62) | -5.04 (-8.08,-2.01) |
| -0.04 (-1.35,1.28) | **N** | -1.23 (-4.16,1.71) | -1.41 (-4.86,2.03) | -1.47 (-4.18,1.24) | -1.56 (-4.96,1.85) | -2.16 (-5.36,1.03) | -2.26 (-5.07,0.56) | -2.31 (-5.47,0.86) | -2.37 (-5.72,0.98) | -2.44 (-5.29,0.42) | -3.69 (-6.73,-0.64) | -4.41 (-7.73,-1.10) | -4.63 (-7.88,-1.38) | -5.08 (-8.39,-1.77) |
| 1.19 (-1.43,3.82) | 1.23 (-1.71,4.16) | **J** | -0.19 (-2.61,2.23) | -0.24 (-1.38,0.89) | -0.33 (-2.69,2.03) | -0.93 (-2.97,1.10) | -1.03 (-2.40,0.35) | -1.08 (-3.08,0.92) | -1.14 (-3.42,1.13) | -1.21 (-2.67,0.25) | -2.46 (-4.26,-0.66) | -3.18 (-5.41,-0.96) | -3.40 (-5.53,-1.27) | -3.85 (-6.06,-1.64) |
| 1.38 (-1.81,4.57) | 1.41 (-2.03,4.86) | 0.19  (-2.23,2.61) | **H** | -0.06 (-2.19,2.08) | -0.14 (-1.99,1.71) | -0.75 (-2.36,0.86) | -0.84 (-3.12,1.43) | -0.89 (-2.25,0.47) | -0.96 (-2.70,0.79) | -1.02 (-3.35,1.30) | -2.27 (-3.98,-0.56) | -3.00 (-5.15,-0.85) | -3.22 (-5.27,-1.16) | -3.66 (-5.81,-1.52) |
| 1.44 (-0.93,3.80) | 1.47 (-1.24,4.18) | 0.24 (-0.89,1.38) | 0.06 (-2.08,2.19) | **I** | -0.08 (-2.15,1.98) | -0.69 (-2.38,1.00) | -0.78 (-1.56,-0.01) | -0.83 (-2.48,0.81) | -0.90 (-2.88,1.07) | -0.97 (-1.88,-0.05) | -2.22 (-3.61,-0.82) | -2.94 (-4.85,-1.03) | -3.16 (-4.96,-1.36) | -3.61 (-5.51,-1.71) |
| 1.52 (-1.62,4.66) | 1.56 (-1.85,4.96) | 0.33 (-2.03,2.69) | 0.14 (-1.71,1.99) | 0.08 (-1.98,2.15) | **K** | -0.61 (-2.12,0.91) | -0.70 (-2.91,1.51) | -0.75 (-2.00,0.50) | -0.82 (-2.48,0.84) | -0.88 (-3.14,1.38) | -2.13 (-3.76,-0.51) | -2.86 (-4.94,-0.77) | -3.07 (-5.06,-1.09) | -3.52 (-5.60,-1.45) |
| 2.13 (-0.78,5.04) | 2.16 (-1.03,5.36) | 0.93 (-1.10,2.97) | 0.75 (-0.86,2.36) | 0.69 (-1.00,2.38) | 0.61 (-0.91,2.12) | **B** | -0.09 (-1.96,1.77) | -0.14 (-1.00,0.71) | -0.21 (-1.58,1.16) | -0.27 (-2.20,1.65) | -1.52 (-2.59,-0.46) | -2.25 (-3.93,-0.57) | -2.47 (-4.03,-0.91) | -2.92 (-4.59,-1.24) |
| 2.22 (-0.27,4.71) | 2.26 (-0.56,5.07) | 1.03 (-0.35,2.40) | 0.84 (-1.43,3.12) | 0.78 (0.01,1.56) | 0.70 (-1.51,2.91) | 0.09 (-1.77,1.96) | **O** | -0.05 (-1.87,1.77) | -0.12 (-2.28,2.05) | -0.18 (-1.09,0.73) | -1.43 (-3.03,0.17) | -2.16 (-4.22,-0.09) | -2.37 (-4.34,-0.41) | -2.82 (-4.88,-0.77) |
| 2.27 (-0.61,5.15) | 2.31 (-0.86,5.47) | 1.08 (-0.92,3.08) | 0.89 (-0.47,2.25) | 0.83 (-0.81,2.48) | 0.75 (-0.50,2.00) | 0.14 (-0.71,1.00) | 0.05 (-1.77,1.87) | **F** | -0.07 (-1.16,1.02) | -0.13 (-2.01,1.75) | -1.38 (-2.42,-0.35) | -2.11 (-3.78,-0.44) | -2.32 (-3.87,-0.78) | -2.77 (-4.43,-1.12) |
| 2.34 (-0.74,5.42) | 2.37 (-0.98,5.72) | 1.14 (-1.13,3.42) | 0.96 (-0.79,2.70) | 0.90 (-1.07,2.88) | 0.82 (-0.84,2.48) | 0.21 (-1.16,1.58) | 0.12 (-2.05,2.28) | 0.07 (-1.02,1.16) | **G** | -0.06 (-2.26,2.13) | -1.31 (-2.81,0.19) | -2.04 (-4.03,-0.05) | -2.26 (-4.14,-0.37) | -2.71 (-4.68,-0.73) |
| 2.40 (-0.13,4.94) | 2.44 (-0.42,5.29) | 1.21 (-0.25,2.67) | 1.02 (-1.30,3.35) | 0.97 (0.05,1.88) | 0.88 (-1.38,3.14) | 0.27 (-1.65,2.20) | 0.18 (-0.73,1.09) | 0.13 (-1.75,2.01) | 0.06 (-2.13,2.26) | **L** | -1.25 (-2.92,0.42) | -1.98 (-4.09,0.14) | -2.19 (-4.21,-0.17) | -2.64 (-4.75,-0.53) |
| 3.65 (0.91,6.40) | 3.69 (0.64,6.73) | 2.46 (0.66,4.26) | 2.27 (0.56,3.98) | 2.22 (0.82,3.61) | 2.13 (0.51,3.76) | 1.52 (0.46,2.59) | 1.43 (-0.17,3.03) | 1.38 (0.35,2.42) | 1.31 (-0.19,2.81) | 1.25 (-0.42,2.92) | **A** | -0.73 (-2.03,0.58) | -0.94 (-2.08,0.20) | -1.39 (-2.68,-0.10) |
| 4.38 (1.34,7.42) | 4.41 (1.10,7.73) | 3.18 (0.96,5.41) | 3.00 (0.85,5.15) | 2.94 (1.03,4.85) | 2.86 (0.77,4.94) | 2.25 (0.57,3.93) | 2.16 (0.09,4.22) | 2.11 (0.44,3.78) | 2.04 (0.05,4.03) | 1.98 (-0.14,4.09) | 0.73 (-0.58,2.03) | **D** | -0.22 (-1.48,1.05) | -0.67 (-2.50,1.17) |
| 4.59 (1.62,7.57) | 4.63 (1.38,7.88) | 3.40 (1.27,5.53) | 3.22 (1.16,5.27) | 3.16 (1.36,4.96) | 3.07 (1.09,5.06) | 2.47 (0.91,4.03) | 2.37 (0.41,4.34) | 2.32 (0.78,3.87) | 2.26 (0.37,4.14) | 2.19 (0.17,4.21) | 0.94 (-0.20,2.08) | 0.22 (-1.05,1.48) | **E** | -0.45 (-2.17,1.27) |
| 5.04 (2.01,8.08) | 5.08 (1.77,8.39) | 3.85 (1.64,6.06) | 3.66 (1.52,5.81) | 3.61 (1.71,5.51) | 3.52 (1.45,5.60) | 2.92 (1.24,4.59) | 2.82 (0.77,4.88) | 2.77 (1.12,4.43) | 2.71 (0.73,4.68) | 2.64 (0.53,4.75) | 1.39 (0.10,2.68) | 0.67 (-1.17,2.50) | 0.45 (-1.27,2.17) | **C** |

**Supplementary Table 11.** League table on adverse events.

Important results will be presented with a yellow background. A: LNG-IUS, B: LNG-IUS+ megestrol acetate, C: LNG-IUS+ medroxyprogesterone acetate, D: LNG-IUS+ general treatment, E: LNG-IUS+ metformin, F: megestrol acetate, G: megestrol acetate +metformin, H: megestrol acetate +tamoxifen, I: medroxyprogesterone acetate, J: medroxyprogesterone acetate+metformin, K:STS inhibitor, L:general treatment, M:mTOR inhibitor, N:mTOR+ megestrol acetate+tamoxifen, O:tamoxifen.


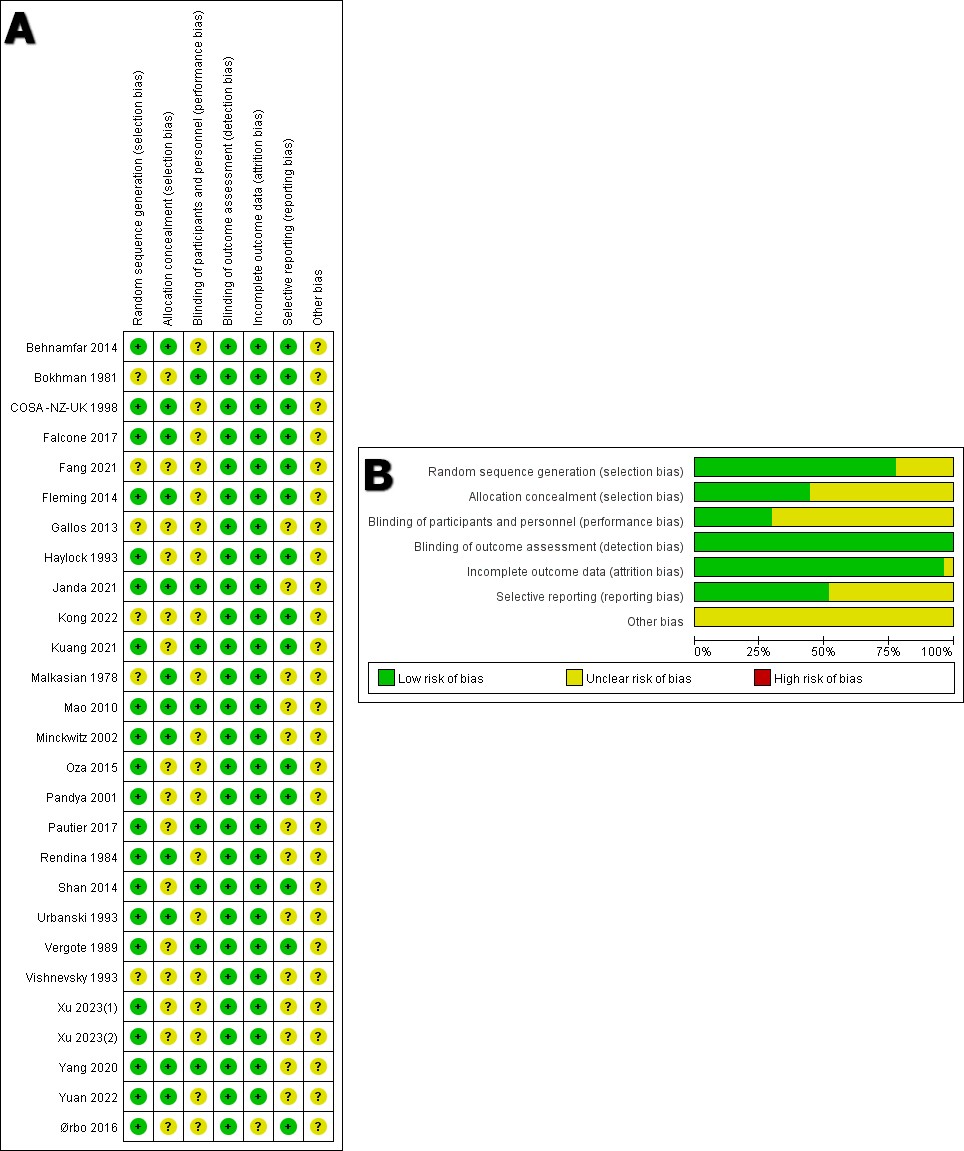


**Supplementary Figure 1.** (**A**) Risk of bias of the included studies, (**B**) Overall risk of bias.


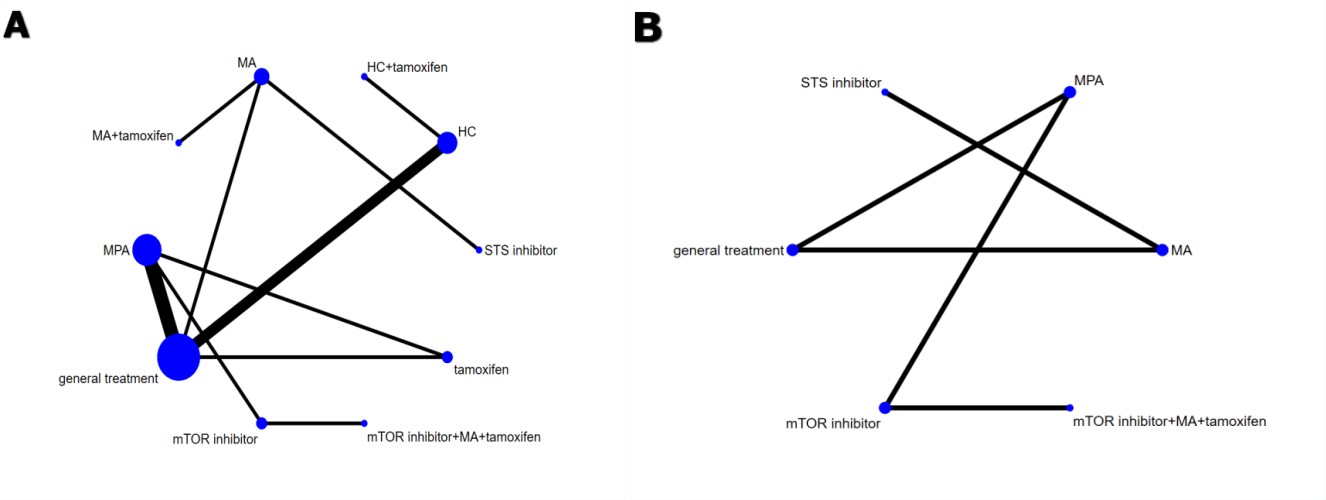


**Supplementary Figure 2.** (**A**) Network map for overall survival (OS), (**B**) Network map for progression-free survival (PFS). HC: hydroxyprogesterone caproate, MA: megestrol acetate, MPA: medroxyprogesterone acetate, STS: steroid sulphatase, mTOR: mammalian target of rapamycin.

**Supplementary Figure 3.** (**A**) Network map for complete response (CR), (**B**) Network map for partial response (PR), (**C**) Network map for objective response rate (ORR), (**D**) Network map for stable disease (SD), (**E**) Network map for progressive disease (PD). MA: megestrol acetate, MPA: medroxyprogesterone acetate, STS: steroid sulphatase, mTOR: mammalian target of rapamycin. LNG-IUS: levonorgestrel-releasing intrauterine system.


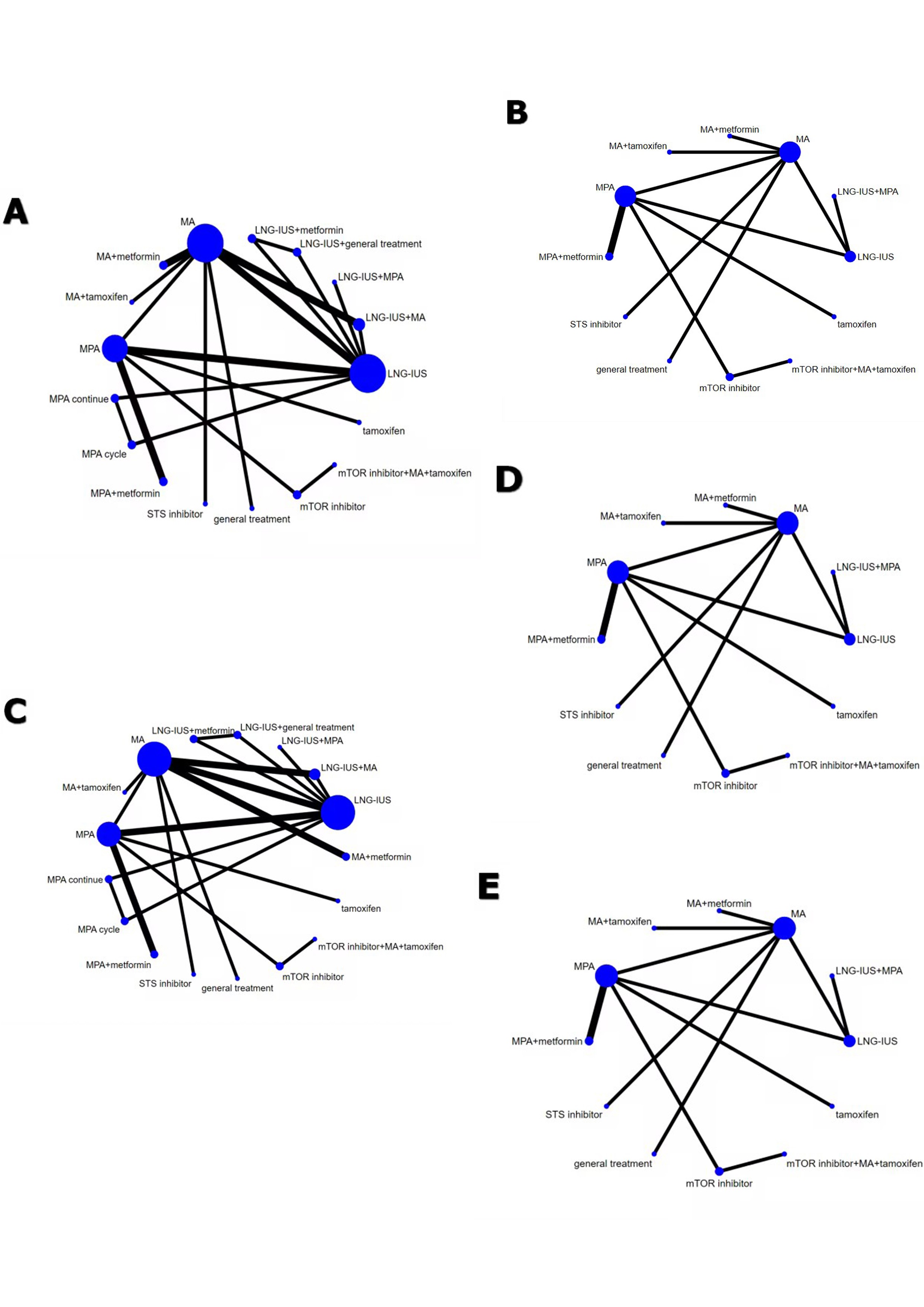


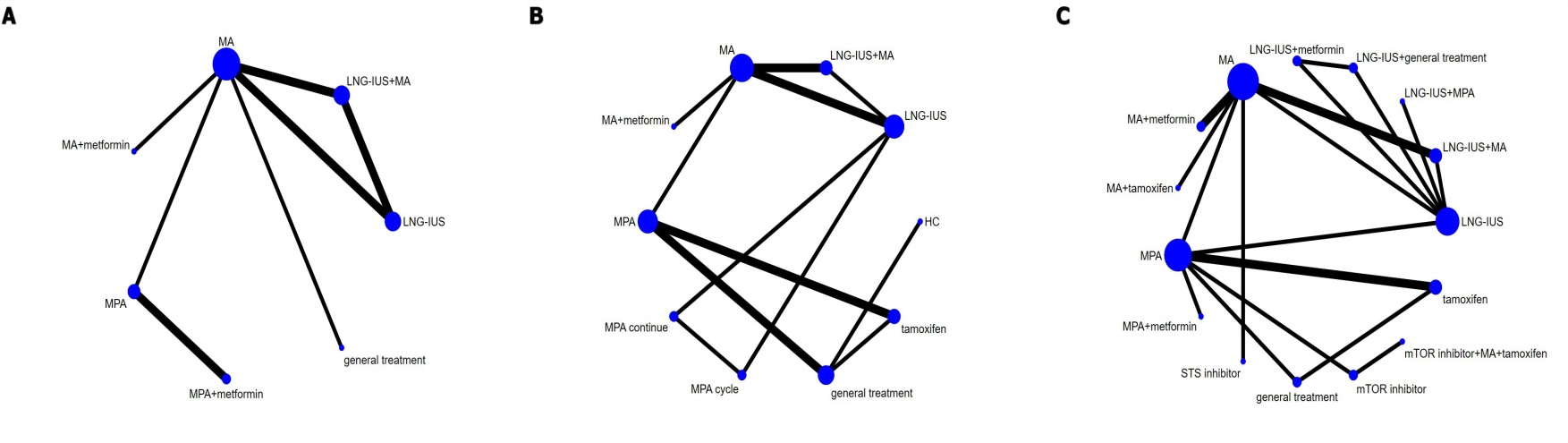


**Supplementary Figure 4.** (**A**) Network map for pregnancy rate, (**B**) Network map for relapse rate, (**C**) Network map for adverse events. MA: megestrol acetate, MPA: medroxyprogesterone acetate, LNG-IUS: levonorgestrel-releasing intrauterine system. HC: hydroxyprogesterone caproate. STS: steroid sulphatase, mTOR: mammalian target of rapamycin.


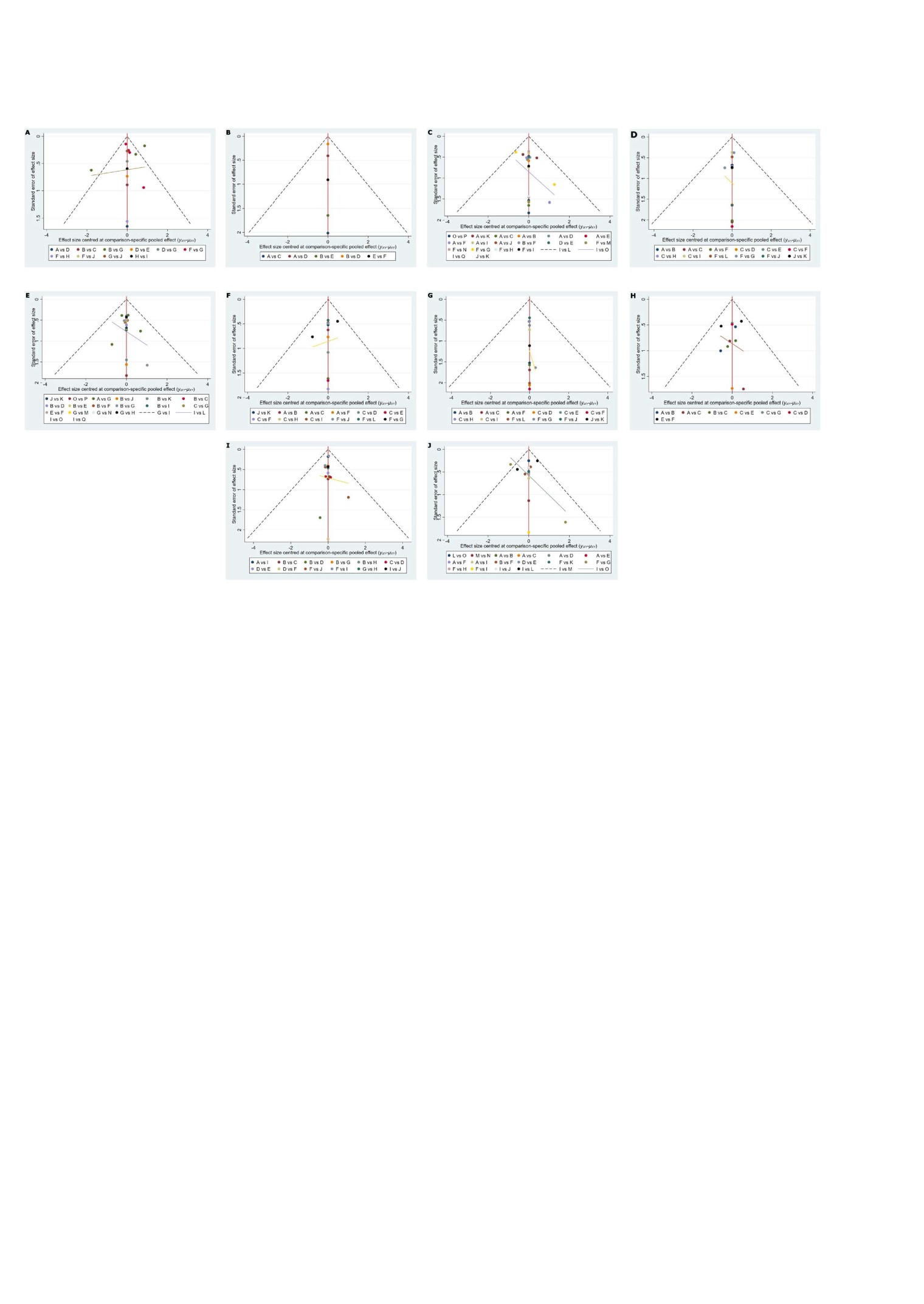
**Supplementary Figure 5.** Funnel plot on publication bias. (**A**) OS, (**B**) PFS, (**C**) CR, (**D**) PR, (**E**) ORR, (**F**) SD, (**G**) PD, (**H**) Pregnancy rate, (**I**) Relapse rate, (J) Adverse events.
